# Early changes in laboratory parameters are predictors of mortality and ICU admission in patients with COVID-19: a systematic review and meta-analysis

**DOI:** 10.1007/s00430-020-00696-w

**Published:** 2020-11-21

**Authors:** Szabolcs Kiss, Noémi Gede, Péter Hegyi, Dávid Németh, Mária Földi, Fanni Dembrovszky, Bettina Nagy, Márk Félix Juhász, Klementina Ocskay, Noémi Zádori, Zsolt Molnár, Andrea Párniczky, Péter Jenő Hegyi, Zsolt Szakács, Gabriella Pár, Bálint Erőss, Hussain Alizadeh

**Affiliations:** 1 Institute for Translational Medicine, University of Pécs Medical School, Pécs, Hungary; 2 János Szentágothai Research Centre, University of Pécs, Pécs, Hungary; 3 First Department of Medicine, University of Szeged, Szeged, Hungary; 4 Doctoral School of Clinical Medicine, University of Szeged, Szeged, Hungary; 5 Department of Anaesthesiology and Intensive Therapy, Poznan University for Medical Sciences, Poznan, Poland; 6 Division of Hematology, First Department of Internal Medicine, University of Pécs Medical School, Pécs, Hungary; 7 Division of Gastroenterology, First Department of Internal Medicine, University of Pécs Medical School, Pécs, Hungary; 8 Heim Pál National Institute of Pediatrics, Budapest, Hungary

**Keywords:** Covid-19, Laboratory, Prognosis, Survival, Mortality, Meta-analysis

## Abstract

Despite the growing knowledge of the clinicopathological features of COVID-19, the correlation between early changes in the laboratory parameters and the clinical outcomes of patients is not entirely understood. In this study, we aimed to assess the prognostic value of early laboratory parameters in COVID-19. We conducted a systematic review and meta-analysis based on the available literature in five databases. The last search was on July 26, 2020, with key terms related to COVID-19. Eligible studies contained original data of at least ten infected patients and reported on baseline laboratory parameters of patients. We calculated weighted mean differences (WMDs) for continuous outcomes and odds ratios (ORs) with 95% confidence intervals. 93 and 78 studies were included in quantitative and qualitative syntheses, respectively. Higher baseline total white blood cell count (WBC), C-reactive protein (CRP), lactate-dehydrogenase (LDH), creatine kinase (CK), D-dimer and lower absolute lymphocyte count (ALC) (WMD_ALC_ = − 0.35 × 10^9^/L [CI − 0.43, − 0.27], *p *< 0.001, *I*^2^ = 94.2%; < 0.8 × 10^9^/L, OR_ALC_ = 3.74 [CI 1.77, 7.92], *p *= 0.001, *I*^2^ = 65.5%) were all associated with higher mortality rate. On admission WBC, ALC, D-dimer, CRP, LDH, and CK changes could serve as alarming prognostic factors. The correct interpretation of laboratory abnormalities can guide therapeutic decisions, especially in early identification of potentially critical cases. This meta-analysis should help to allocate resources and save lives by enabling timely intervention.

**Electronic supplementary material:**

The online version of this article (10.1007/s00430-020-00696-w) contains supplementary material, which is available to authorized users.

## Introduction

Coronavirus disease-19 (COVID-19) is a novel coronavirus infection caused by the novel Severe Acute Respiratory Syndrome Coronavirus 2 (SARS-CoV-2), which was first detected in Wuhan, China, in December 2019 after a series of pneumonia cases of unknown aetiology had emerged [1]. On 11 March 2020, WHO declared the rapid spread of this virus a pandemic [2]. Since the initial detection of the virus, more than 25,000,000 cases of COVID-19 have been confirmed worldwide with over 850,000 fatal cases [3].

In some patients, symptoms of severe respiratory infection can occur with rapidly developing acute respiratory distress syndrome and other serious complications, which may be followed eventually by multiple organ failure and death. Therefore, early diagnosis and timely treatment of critical cases are crucial.

Despite some knowledge of the clinicopathological features of COVID-19, the correlation of changes in laboratory parameters and the prognosis of patients with COVID-19 is still unclear. However, studies on COVID-19 cases have shown that increased levels of white blood cells (WBC), decreased numbers of lymphocytes, especially CD8 + cells, increased levels of lactate-dehydrogenase (LDH), creatine kinase (CK), C-reactive protein (CRP), D-dimer, and levels of pro-inflammatory cytokines are associated with more severe inflammation and extensive lung damage with higher rates of admission to intensive care unit (ICU) and mortality [4]. A better understanding of early prognostic clinical laboratory parameters could save many lives by enabling timely intervention and better resource allocation since ICU capacity is limited in most countries. In this meta-analysis, we aimed to explore the significance of changes in the laboratory parameters and assessed the correlation between clinical laboratory data and the clinical outcomes of patients with COVID-19.

## Methods

This systematic review with meta-analysis is reported in accordance with the Preferred Reporting Items for Systematic Reviews and Meta-Analyses Statement [5]. The review protocol was registered on PROSPERO (CRD42020176836).

### Search strategy

The systematic literature search was conducted in MEDLINE (via PubMed), Embase, Cochrane Library (CENTRAL), Scopus, and Web of Science for studies published from 1st January 2020 to 9th April 2020. The following search terms were used: (”covid 19”) OR (“Wuhan virus”) OR (“coronavirus”) OR (“2019 nCoV”) OR (“SARS-cov-2”). There was no restriction on the language of the records.

### Selection and eligibility criteria

We selected clinical studies reporting on at least ten confirmed SARS-CoV-2 infected patients (based on the WHO case definition) and their laboratory findings. Studies were included in the systematic review of data on at least one of the following variables could be extracted: total white blood cell count (WBC), absolute lymphocyte count (ALC), absolute neutrophil count (ANC), platelet count, absolute basophil count, absolute eosinophil count (AEC), absolute monocyte count (AMC), C-reactive protein (CRP), haemoglobin, ferritin, lactate dehydrogenase (LDH), creatine kinase (CK), procalcitonin (PCT), fibrinogen, D-dimer, and any interleukins or lymphocyte subsets (CD3 + , CD4 + , CD8 +). The titles, abstracts, and full texts of the studies were screened by four independent review authors in pairs based on predefined criteria. The decision to include a study in the meta-analysis was based upon the assessment of the two reviewers and, if necessary, by a third reviewer for the resolution of any disagreements. Reference lists in the included studies and reviews on this topic were searched for additional studies. Publications citing the included studies were screened in the Google Scholar academic search engine too. Those studies that had either proven or suspected overlapping populations were included only in the systematic review part of this paper. To clarify these overlaps, we tried to contact the corresponding authors. Studies with more than 10% unclosed cases were excluded.

### Data extraction

Four review authors independently extracted data into a standardized data collection form. The following data were extracted from each eligible article: first and second author, publication year, study site, study design, gender, age, and the means, standard deviations, medians, ranges, and interquartile ranges (IQR) of the laboratory values and specific thresholds with the corresponding intensive care requirement and mortality ratio. Data extraction was validated by a fifth review author. Discrepancies were resolved by a third party.

### Risk of bias assessment

Based on the recommendation of the Cochrane Prognosis Methods Group, the QUIPS tool was applied by two independent authors for assessing the risk of bias in the studies included. Any disagreement was resolved based on consensus [6].

### Statistical analysis

Pooled mean difference (weighted mean difference, WMD) was calculated for continuous outcomes and pooled odds ratios (ORs) were calculated for dichotomous outcomes. Random effect model was applied to all of the analyses with DerSimonien-Laird estimation. Statistical heterogeneity was analysed using the *I*^2^ the *χ*^2^ tests to obtain probability values: *p *< 0.01 was defined as indicating significant heterogeneity. Where mean with standard deviation was not reported for any of the outcomes, they were estimated from median, interquartiles and range using the method of Wan (2014) [7]. We performed separate analyses for mortality based on the clinical characteristics of the study population: one for all hospitalized COVID-19 patients (the “mixed” population) and the other for only critically ill COVID-19 patients. Small study effect was evaluated by visual assessment of funnel plot asymmetry and by Egger’s test were more than ten studies where available. Statistical analyses were performed with Stata 15 SE (Stata Corp). In the case of potentially overlapping study populations, data from the study with higher participant numbers were used for each outcome. ORs were calculated where raw data were available, however, only those meta-analyses were interpreted where at least three non-overlapping studies were available, as required.

## Results

**Fig. 1:**
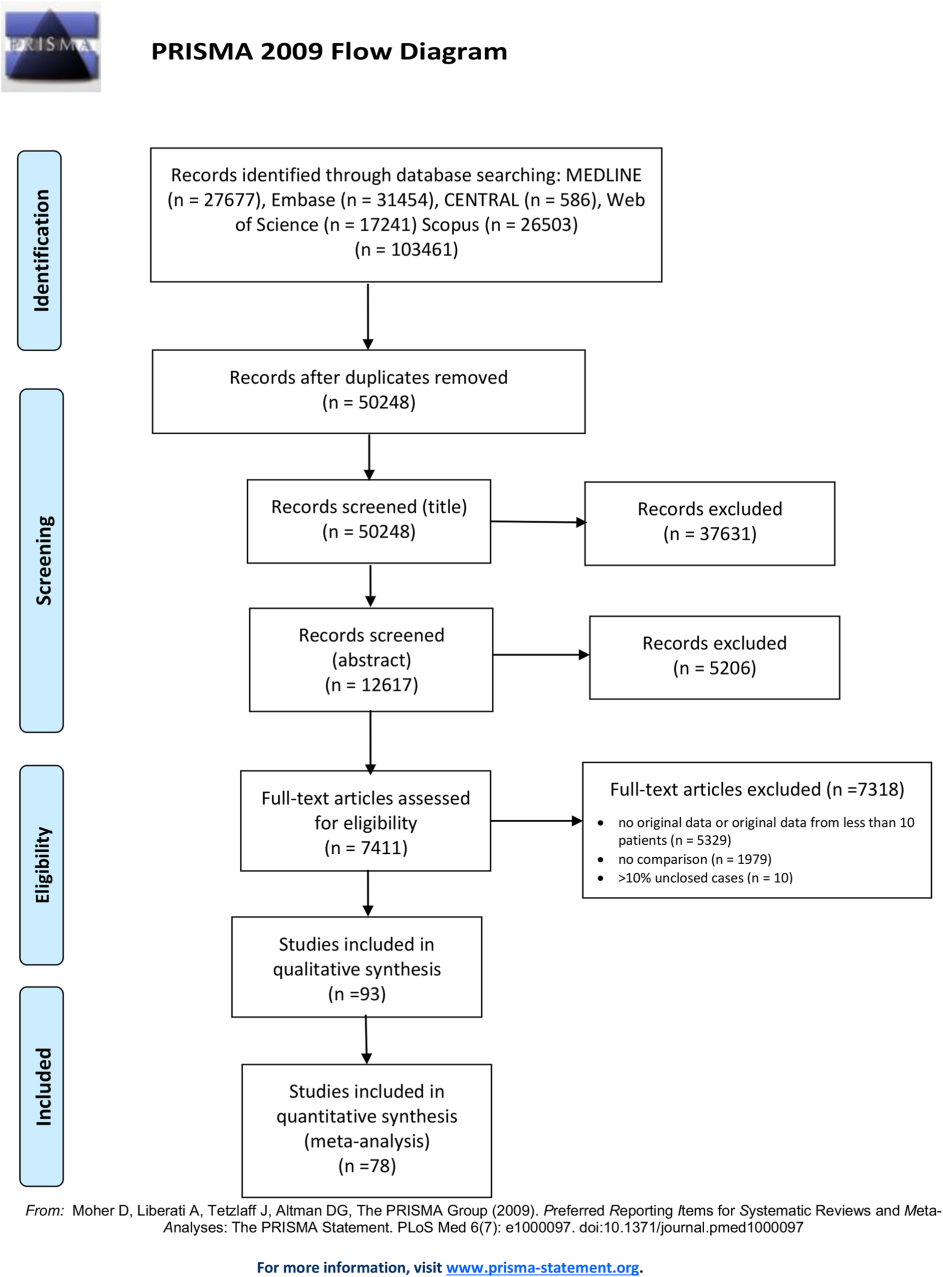
PRISMA Flow Diagram showing the systematic search and selection process

The results of our search and selection are detailed in the PRISMA-Flowchart shown in Fig. 1. Our systematic search yielded 93 eligible studies from 16 countries. We summarize the characteristics of the included studies in Supplementary Table 1. Out of these, fifty-six studies reported on the association of laboratory parameters and mortality. [8–63]. Of these, forty-eight studies reported on 25,901 patients with all levels of disease severity (the “mixed” population), and eleven other studies discussed critically ill cases with an overall patient number of 2804. Forty-one studies with 11,935 patients comparing those with and without ICU requirement have also been included in this review [8, 19, 26, 29, 43, 64–100].

The incidence of mortality ranged from 6.25 to 61.5% in the mixed population and from 22.35 to 71.19% in the critically ill population. While the prevalence of ICU requirements ranged from 8.76 to 70.59%.

Results of the qualitative and quantitative synthesis are summarized in Supplementary Tables 2, 3, 4.

### Weighted mean differences

**Fig. 2:**
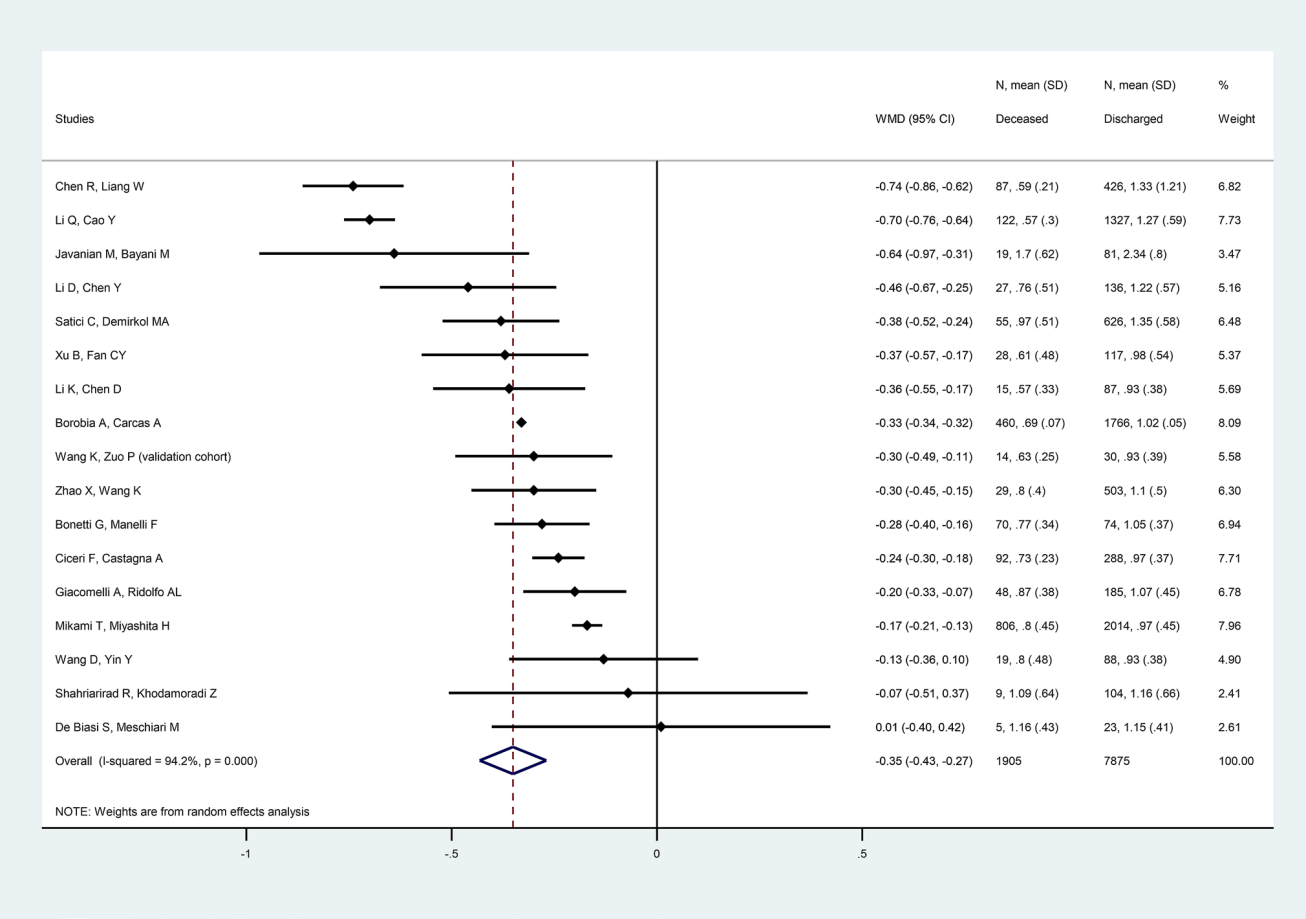
Forest plot representing that decreased baseline absolute lymphocyte count was associated with increased mortality

Pooled analyses showed that among all COVID-19 patients mortality was associated with increased baseline WBC (WMD = 2.35 × 10^9^/L [CI 1.96, 2.83], *p *< 0.001, *I*^2^ = 64.5%), ANC (WMD = 2.67 × 10^9^/L [CI 2.12, 3.21], *p *< 0.001, *I*^2^ = 71.7%), CRP (WMD = 65.65 mg/L [CI 42.79, 87.50], *p *< 0.001, *I*^2^ = 99.4%), LDH (WMD = 203.79 U/L [CI 151.86, 255.71], *p *< 0.001, *I*^2^ = 95.2%), PCT (WMD = 0.38 ng/mL [CI 0.30, 0.47], *p *< 0.001, *I*^2^ = 91.8%), fibrinogen (WMD = 0.32 g/L [CI 0.13, 0.50], *p *= 0.001, *I*^2^ = 52.4%), D-dimer (WMD = 1.31 mg/L [CI 1.05, 1.57], *p *< 0.001, *I*^2^ = 84.5%), ferritin (WMD = 550.20 μg/L [CI 347.97, 752.43], *p *< 0.001, *I*^2^ = 15.8%), CK (WMD = 77.59 U/L [CI 55.31, 99.86], *p *< 0.001, *I*^2^ = 81.4%) and IL-6 (WMD = 84.26 pg/mL [CI 49.23, 119.30], *p *< 0.001, *I*^2^ = 97.5%). In the same population, decreased baseline ALC (WMD = − 0.35x10^9^/L [CI − 0.43, − 0.27], *p *< 0.001, *I*^2^ = 94.2%), CD3 + lymphocyte count (WMD = − 329.71 cell/μL [CI − 370.82, − 288.59], *p *< 0.001, *I*^2^ = 60.1%), CD4 + lymphocyte count (WMD = − 164.24 cell/μL [CI − 190.51, − 137.97], *p *< 0.001, *I*^2^ = 67.0%), CD8 + lymphocyte count (WMD = − 115.45 cell/μL [CI − 130.61, − 100.30], *p *< 0.001, *I*^2^ = 55.7%), AEC (WMD = − 0.02 × 10^9^/L [CI − 0.03, − 0.01], *p *= 0.003, *I*^2^ = 74.6%), AMC (WMD = − 0.05 × 10^9^/L [CI − 0.08, − 0.03], *p *< 0.001, *I*^2^ = 0.0%), and platelet count (WMD = − 25.66 × 10^9^/L [CI − 35.56, − 15.76], *p *< 0.001, *I*^2^ = 81.8%) was associated with increased mortality. (Fig. 2) We have not found significant association between baseline IL-1 and mortality among all COVID-19 patient.

Pooled analyses found that among all critically ill COVID-19 patients, mortality was associated with increased baseline LDH (WMD = 129.34 U/L [CI 67.73, 190.94], *p *< 0.001, *I*^2^ = 34.1%), increased CRP (WMD = 45.36 mg/L [CI 23.50, 87.50], *p *< 0.001, *I*^2^ = 35.3%), and decreased platelet levels (WMD = − 30.19 × 10^9^/L [CI − 44.88, − 15.50], *p *< 0.001, *I*^2^ = 0.0%). We have not identified significant baseline difference between deceased and discharged critically ill patients regarding WBC, ALC, PCT, and D-dimer levels.

Pooled analyses revealed that the following baseline laboratory parameters were higher in patients who required intensive care compared those did not: WBC (WMD = 1.53 × 10^9^/L [CI 1.04, 2.02], *p *< 0.001, *I*^2^ = 68.8%), ANC (WMD = 2.47 × 10^9^/L [CI 1.71, 3.23], *p *= 0.037, *I*^2^ = 75.2%), CRP (WMD = 65.65 mg/L [CI 42.79, 87.50], *p *< 0.001, *I*^2^ = 99.4%), LDH (WMD = 190.91 U/L [CI 129.40, 252.42], *p *< 0.001, *I*^2^ = 90.4%), PCT (WMD = 0.21 ng/mL [CI 0.05, 0.37], *p *= 0.008, *I*^2^ = 95.6%), CK (WMD = 54.07 U/L [CI 28.37, 79.77], *p *< 0.001, *I*^2^ = 35.2%), fibrinogen (WMD = 1.04 g/L [CI 0.66, 1.43], *p *< 0.001, *I*^2^ = 0.0%), D-dimer (WMD = 0.77 mg/L [CI 0.50, 1.04], *p *= 0.007, *I*^2^ = 81.1%), ferritin (WMD = 328.28 μg/L [CI 181.58, 474.99], *p *< 0.001, *I*^2^ = 15.8%), and IL-6 (WMD = 26.67 pg/mL [CI 15.98, 37.35], *p *< 0.001, *I*^2^ = 0.0%). Intensive care requirement was also associated with decreased baseline ALC (WMD = − 0.30 × 10^9^/L [CI − 0.37, − 0.23], *p *< 0.001, *I*^2^ = 87.0%), CD3 + lymphocyte count (WMD = − 322.56 cell/μL [CI − 589.00, − 55.54], *p *= 0.018, *I*^2^ = 83.5%), CD4 + lymphocyte count (WMD = − 142.98 cell/μL [CI − 242.12, − 43.85], *p *= 0.005, *I*^2^ = 82.2%), CD8 + lymphocyte count (WMD = − 186.52 cell/μL [CI − 254.84, − 118.21], *p *< 0.001, *I*^2^ = 73.3%), and haemoglobin (WMD = − 7.39 g/L [CI − 11.65, − 3.14], *p *= 0.001, *I*^2^ = 64.1%). No significant association was found between intensive care requirement and baseline AMC, platelet count.

### Odds ratios

**Fig. 3:**
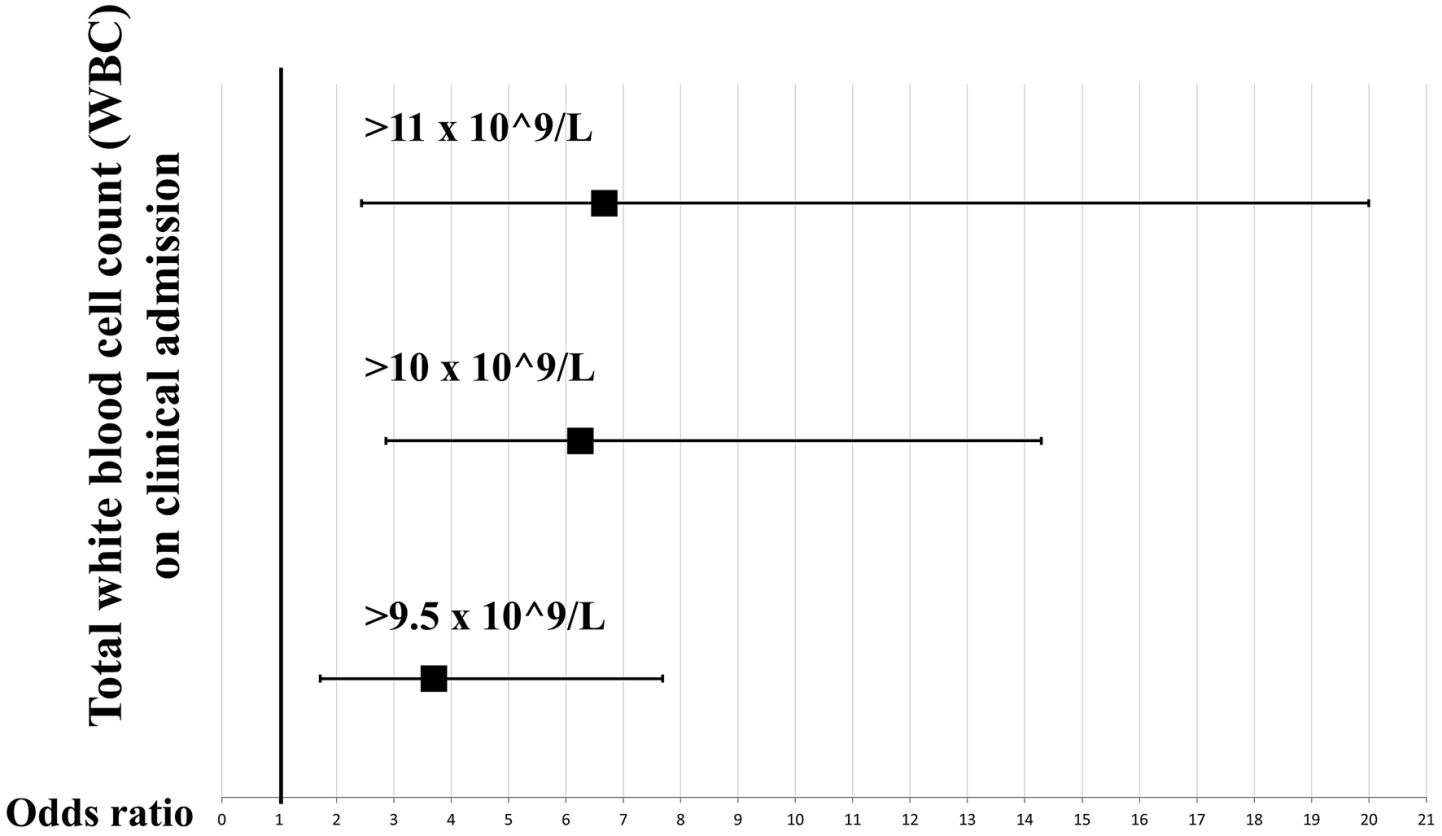
Odds ratios suggest a stepwise increase in risk for mortality parallel with the increase of the total white blood cell threshold

Among all COVID-19 patients, increased on admission total WBC was found to be a risk factor for mortality (> 9.5 × 10^9^/L, OR = 3.7 [CI 1.72, 7.69], *p *= 0.001, *I*^2^ = 0.0%; > 10.0 × 10^9^/L, OR = 6.25 [CI 2.86, 14.29], *p *< 0.001, *I*^2^ = 85.2%) and intensive care requirement (> 9.5 × 10^9^/L, OR = 4.52 [CI 1.95, 10.52], *p *< 0.001, *I*^2^ = 26.8%; > 10.0 × 10^9^/L, OR = 2.64 [CI 1.22, 5.71], *p *= 0.014, *I*^2^ = 61.3%). These results suggest a stepwise increase in risk for mortality in parallel with the increase of the total WBC threshold. This is depicted on Fig. 3. Furthermore, low baseline WBC was associated with decreased mortality (< 4.0 × 10^9^/L, OR = 0.38 [CI 0.20, 0.72], *p *= 0.003, *I*^2^ = 40.6%) and lower risk for intensive care requirement (< 3.5 × 10^9^/L, OR = 0.42 [CI 0.18, 0.96], *p *= 0.039, *I*^2^ = 0.0%).

**Fig. 4:**
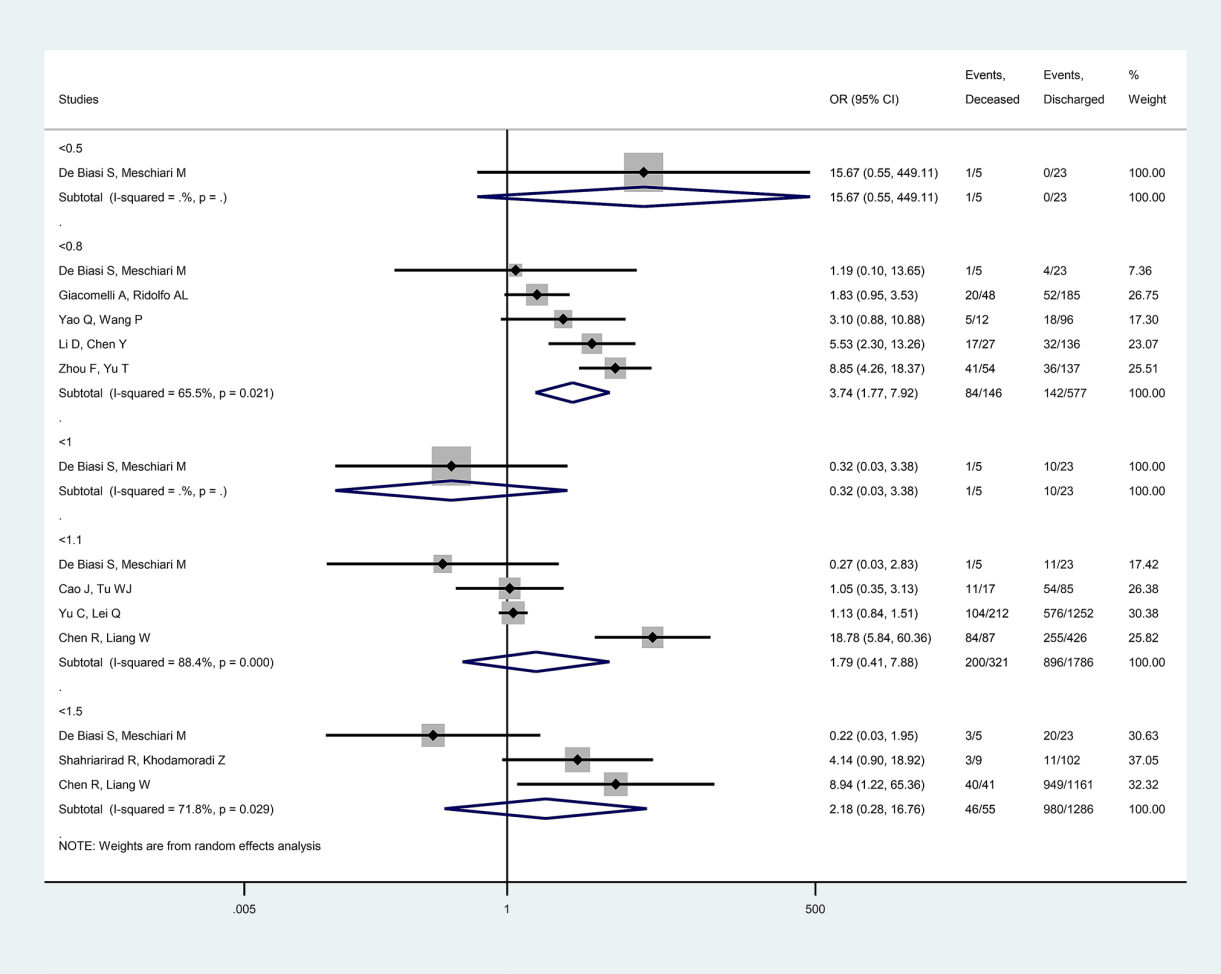
Forest plot representing that low absolute lymphocyte count carries and increased risk for mortality

Low ALC on clinical admission was a risk factor for mortality (< 0.8 × 10^9^/L, OR = 3.74 [CI 1.77, 7.92], *p *= 0.001, *I*^2^ = 65.5%) and intensive care requirement (< 1.0 × 10^9^/L, OR = 4.54 [CI 2.58, 7.95], *p *< 0.001, *I*^2^ = 26.8%; < 1.1 × 10^9^/L, OR = 2.64 [CI 1.49, 4.70], *p *= 0.001, *I*^2^ = 36.4%) among all COVID-19 patients. (Fig. 4).

Increased baseline ANC was found to be a risk factor for intensive care requirement (> 6.3 × 10^9^/L, OR = 2.32 [CI 1.23, 4.37], *p *= 0.009, *I*^2^ = 0.0%). We could not carry out a meta-analysis for any threshold regarding mortality, however individual studies support its role as a risk factor for mortality [23, 34, 49].

Assessment of low platelet on admission as a risk factor for mortality provided inconsistent results. Although baseline platelet level under 125 × 10^9^/L was associated with a significantly higher risk for mortality among all COVID-19 patients, on admission platelet level below 100 × 10^9^/L and 150 × 10^9^/L did not show significant results. We did not find any threshold that is associated with increased risk for intensive care requirement.

Evaluation of increased CRP showed that baseline level over 10 mg/L and 100 mg/L is associated with increased mortality (OR = 4.84 [CI 1.49, 15.69], *p *= 0.009, *I*^2^ = 45.8%; OR = 2.49 [CI 1.42, 4.35], *p *= 0.001, *I*^2^ = 14.7%, respectively), however, the analysis regarding the threshold of 50 mg/L was not significant, which makes these results inconsistent. In case of intensive care requirement, baseline level over 10 mg/L was found to be a risk factor (OR = 3.85 [CI 1.21, 12.22], *p *= 0.022, *I*^2^ = 55.4%).

On admission LDH over 250 U/L was found to be a risk factor both mortality (OR = 10.88 [CI 4.48, 26.39], *p *< 0.001, *I*^2^ = 0.0%) and intensive care requirement (OR = 9.44 [CI 4.412, 24.02], *p *< 0.001, *I*^2^ = 0.0%).

Baseline procalcitonin level over 0.05 ng/mL was not a risk factor for mortality, however, we found increased risk over the threshold of 0.50 ng/mL (OR = 11.97 [CI 4.75, 30.16], *p *< 0.001, *I*^2^ = 59.4%). The same thresholds provided non-significant results regarding intensive care requirement.

Increased D-dimer level on admission was found to be a risk factor for mortality (> 0.50 mg/L, OR = 4.30 [CI 1.55, 11.98], *p *= 0.005, *I*^2^ = 83,7; > 1.0 mg/L, OR = 6.63 [CI 3.62, 12.14], *p *< 0.001, *I*^2^ = 45.1%) and intensive care requirement (> 0.50 mg/L, OR = 3.37 [CI 1.90, 5.95], *p *< 0.001, *I*^2^ = 0.0%).

On admission CK level over 185 U/L was associated with increased mortality (OR = 3.14 [CI 1.87, 5.27], *p *< 0.001, *I*^2^ = 0.0%). We could not carry out a meta-analysis for any threshold regarding intensive care requirement, however, individual studies support the role of increased CK as a risk factor [79, 85, 98].

There was no common threshold for any laboratory parameters with more than three non-overlapping studies, therefore, we were unable to calculate ORs for mortality among critically ill COVID-19 patients. ORs for mortality and intensive care requirements are summarized in Supplementary Table 3.

### Risk of bias assessment and publication bias

Results of risk of bias assessments and evaluation of small-study effect are summarized in Supplementary Figures and among limitations of this study.

## Discussion

In this meta-analysis, we have assessed the correlations between changes in laboratory parameters and the outcomes of patients with COVID-19. In doing so, we have identified many laboratory parameters that could be crucial for the timely identification of patients at higher risk of adverse outcomes.

This is the most comprehensive meta-analysis that assesses associations between on-admission laboratory parameters and mortality, as well as intensive care requirement. Compared with previous meta-analyses, [101–129]. our work contains the widest coverage of laboratory parameters in this topic with the largest sample size, from 16 different countries. To the best of our knowledge, this study is the only meta-analysis which assessed all potential thresholds for the investigated parameters regarding mortality and intensive care requirement. We also analysed the role of early laboratory parameters in an important subgroup: in patients who were critically ill on admission and had consequently higher mortality. We strictly evaluated all studies to avoid pooling studies with potentially overlapping population and unclosed cases.

Our study provides further evidence for a remarkable early prognostic value of ALC in COVID-19 since we found that low absolute lymphocyte levels on admission present a significant risk for critical illness and mortality, but probably with different thresholds. In addition to these early changes, it has been reported that absolute lymphocyte counts remained low for an additional few days in survivors and improved later, while in non-survivors, lymphopenia did not improve and in the majority of cases this further progressed [33, 62]. Lymphocyte depletion might be explained by direct viral damage or by the imbalance of inflammatory mediators [130].

We also found that CD3 + , CD4 + and CD8 + cells were greatly decreased in non-survivors [4, 85]. Importantly, these lymphocyte subsets play a role in viral clearance, reducing overreaction of the immune system [131],. and developing long-term immunity including that achieved after vaccination [130, 132].

We have noted that patients with a higher total WBC on admission had a poorer prognosis, while low total WBC levels were found to be a protective factor. Higher total WBC values are probably due mainly to increased levels of neutrophils [133]. In support of this idea, higher neutrophil counts also “predisposed” patients to unfavourable disease outcomes [134]. In light of our current knowledge, this might not be surprising since neutrophils are responsible for the production of pro-inflammatory mediators. Overproduction of these mediators, the so-called cytokine storm, has been suggested as a major cause of critical illness and mortality in COVID-19 [135].

It is important to note that increased levels of proinflammatory mediators such as CRP, fibrinogen and IL-6 were associated with worse outcomes. In agreement with previous studies, we found higher ferritin levels in non-survivors and critically ill patients. The laboratory profile in COVID-19 indicates hyperinflammation and may resemble secondary haemophagocytic lymphohistiocytosis (sHLH). However, other diagnostic criteria of sHLH have been rarely observed in COVID-19 [136–138].

This knowledge may help to identify therapeutic targets to minimize the cytokine storm. In addition, identifying those at higher risk of a cytokine storm is essential for treating them appropriately in advance [139].

Procalcitonin is not typically increased in viral infections; thus its elevated level at admission may not seem to be a significant finding in patients with COVID-19. Interestingly, according to our results, increased PCT levels have a predictive value for mortality, but not for intensive care requirement. An increase in its level might be associated with worse prognosis, possibly because of a bacterial superinfection, which could contribute to a rapid deterioration in the clinical course of disease towards multiorgan failure and death [140].

Compared to SARS-CoV, low platelet levels in COVID-19 are less common findings on admission. [141]. Although we found lower platelet levels in deceased patients compared to discharged ones, our pooled analyses did not indicate a clear prognostic role for platelet counts. However, studies found decreasing levels of platelet in patients are associated with adverse outcomes during the hospital stay [142, 143]. Thus, continuous monitoring of platelet counts may be required, even if its level initially gives no cause for concern.

Elevated D-dimer level is a typical sign of coagulation abnormalities in COVID-19 [144]. In our meta-analysis, increased D-dimer level was associated with worse prognosis in every comparison, except for the mean baseline D-dimer level between deceased and discharged critically ill patients (*p *= 0.149). However, the interpretation of these finding is uncertain since D-dimer levels can depend on several factors, including the presence of comorbidities or inflammatory processes [145].

The general indicators of tissue damage, elevated LDH and CK, were also associated with unfavourable outcomes in our meta-analysis, but none of these two laboratory parameters are specific for a special condition.

The underlying causes of the laboratory abnormalities are not entirely understood. Thus, further studies, including animal experiments, histological and pathological examinations, and clinical trials might give insight and identify potential therapeutic targets. More studies are required to further specify the thresholds applicable in clinical practice and resolve the contradiction in the role of certain biomarkers. Besides static values, the dynamics of laboratory parameters would worth further studying.

This meta-analysis has some limitations. Because of the nature of studies included, selection bias can occur, particularly in the case of parameters that are not routinely measured [62]. There was considerable heterogeneity in some analyses. Additionally, because of some studies with a high risk of bias, our results need to be interpreted cautiously. High risk of bias among studies mainly resulted from the significant differences in baseline characteristics of patients. Patients with advanced age and comorbidities are at higher risk both for more severe COVID-19 and for laboratory abnormalities. Conversion of medians to means could also distort our results. The visual assessment of funnel plots and Egger’s tests detected small-study effects in most of the analyses concerning WMD analyses.

In conclusion, we have shown that laboratory parameters on admission serve as important and early prognostic factors. These findings should help to allocate resources and potentially to save lives by enabling timely intervention.

## Electronic supplementary material

Below is the link to the electronic supplementary material.

Supplementary material 1 (PDF 1653 kb)

## Data Availability

The data that support the findings of this study are available from the corresponding author, [A.H.], upon reasonable request.
