# Supplementary material for: Early changes in laboratory parameters are predictors of mortality and ICU admission in patients with COVID-19: a systematic review and meta-analysis
Source: Med Microbiol Immunol. 2020 Nov 21;210(1):33–47. doi: 10.1007/s00430-020-00696-w (PMC7679241; doi:10.1007/s00430-020-00696-w)
Supplement: Supplementary file 1 — Supplementary material 1 (PDF 1653 kb) [file 430_2020_696_MOESM1_ESM.pdf]

Study name	Study design	Study site	Period of enrolment	Sample size	Characteristics of participants	Prevalence of mortality/ICU admission (%)
**Studies assessing the risk for mortality in all COVID-19 patients**						
Al-Samkari H, Leaf RK	retrospective cohort	5 hospitals in Boston, Massachusetts, USA	1 March-5 April, 2020	252	n.r.	11.51
Asghar MS, Kazmi, SJH	retrospective cohort	Karachi, Pakistan	March-April, 2020	100	mean age: 52.58±15.68, 31% female	22.00
Barman HA, Atici A	retrospective cohort	3 hospitals in Istanbul, Turkey	20 March - 20 April, 2020	607	age n.r., 45% female	16.97
Bhargava A, Fukushima EA	retrospective observational study	St John Hospital, Detroit, Michigan, USA	8 March-8 April, 2020	197	mean age: 60.6±16.2, 47.7% female	n.r.
Bazzan M, Montaruli B	n.r.	Turin, Italy	n.r.	88	age n.r., 31.8% female	10.23
Bonetti G, Manelli F	retrospective cohort	Emergency Department of the Valcamonica Hospital, Esine, Brescia, Lombardy, Italy	1 March-30 March, 2020	144	age n.r., 33.3% female	48.61
Borobia A, Carcas A	retrospective cohort	La Paz University Hospital, Madrid, Spain	25 February-19 April, 2020	2226	median age 61 (IQR 46-78), 51.8% female	20.66
Cao J, Tu WJ	retrospective cohort	Zhongnan Hospital, Wuhan, China	3 January - 1 February, 2020	102	median age 54 (IQR 37-67), 48% female	16·7
Chen L, Yu J	retrospective cohort	5 hospitals in China	20 January- 4 April, 2020	1859	median age 59 (IQR 45-68), 49,76 % female	11.12
Chen R, Liang W	retrospective cohort	575 hospitals in China	until 31 January, 2020	1590	n.r.	3.14
Chen R, Sang L	retrospective cohort	Wuhan, China	until 22 March, 2020	548	mean age 56±14.5, 42.9% female	18.79
Chen X, Zhao B	retrospective cohort	General Hospital of Central Theater Command, PLA, China	1 February-19 February, 2020	48	mean age 64.6±18.1, 22.9% female	6.25
Ciceri F, Castagna A	retrospective cohort	San Raffaele Hospital, Milan, Italy	25 February- 24 March, 2020	410	median age 76 (IQR 67-82) 27.1 % female	24.61
De Biasi S, Meschiari M	case-control	Infectious Diseases Clinics of the University Hospital in Modena, Italy	12 March-30 March, 2020	29	mean age 61.89±14, 17.24% female	17.24
Fan JL, Wang H	retrospective cohort	Zhongnan Hospital of Wuhan University in Wuhan, China	18 January-8February, 2020	21	mean age 62.5±12.6, 47.7 % female	19.05
Galloway JB, Norton S	observational cohort	King's College Hospital and Princess Royal University Hospital, London, UK	1 March- 17 April, 2020	1157	median age: 71 (IQR 57,82), 42.4% female	21.10
Gan J, Li J	retrospective case-control	Tongji Hospital, Wuhan, China	6 February - 8 March, 2020	95	median age 65 (IQR 56-76), 39% female	41.05
Giacomelli A, Ridolfo AL	prospective cohort	Luigi Sacco Hospital in Milan, Italy	21 February-19 March, 2020	233	median age 61 (IQR 50-72), 30.9% female	20.60
Javanian M, Bayani M	retrospective cohort	Ayatollah Rohani, Shahid Beheshti and Yahyanejad hospitals, Babol, Iran	25 February- 12 March, 2020	100	mean age 60.12±13.87, 49% female	19.00
Li D, Chen Y	retrospective cohort	West China Hospital, Sichuan University, Chengdu, China	31 January-18 February, 2020	163	n.r.	16.56
Li K, Chen D	retrospective cohort	Tongji Hospital, Wuhan, China	31 January- 25 March, 2020	102	median age 57 (IQR 45-70), 42% female	14.71
Li L, Yang L	retrospective cohort	Wuhan Union Hospital, Wuhan, China	1 January- 22 February, 2020	93	mean age 51±17.5, 44% female	26.88
Li Q, Cao Y	retrospective cohort	7 centers of 5 hospitals in China	20 January- 4 April, 2020	1449	median age 57 (IQR 42-66), 49% female	8.42
Li Y, Peng S	retrospective cohort	Thoracic Surgery Department, Tongji	1 January - 20 February, 2020	25	infected health car staff with a median	20.00

		Hospital, Wuhan, China			age of 32 (22-51) and infected hospitalized patients with a median age of 61 (range 51-69); 65% female	
Liu Y, Sun W	retrospective cohort	the Central Hospital of Wuhan, China	2 January- 1 March, 2020	383	median age: 46 (IQR (34–61), 57.7% female	12.8
Long H, Nie L	retrospective cohort	Tianyou Hospital affiliated to the Wuhan University of Science and Technology, Wuhan, China	18 January- 5 March, 2020	75	age n.r., 46.7 % female	30,67
Luo M, Liu J	retrospective cohort	Wuhan Pulmonary Hospital and Tongji Hospital, Huazhong University of Science and Technology, China	9 January- 31 March, 2020	1018	median age 61 (IQR 49-69), 48.8 % female	19.74
Mikami T, Miyashita H	retrospective cohort	8 hospitals in New York, USA	13 March - 17 April, 2020	2820	age n.r., 42.9% female	28.58
Omrani-Nava V, Maleki I	case controll	Mazandaran University of Medical Sciences, Iran	February-March, 2020	93	mean age: 56.3±15.2, 45.2% female	n.r.
Price-Haywood EG, Burton J	retrospective cohort	Ochsner Health, New Orleans, Louisiana, USA	1 March-11 April, 2020	3481	age n.r., 60% female	n.r.
Rivera-Izquierdo M, Valero-Ubierna MDC	retrospective case-series	Hospital Universitario, Clínico San Cecilio, Granada, Spain	16 March-10 April, 2020	238	mean age: 64.7±15.4, 45% female	25.6
Ruan Q, Yang K	retrospective cohort	Jinyintan and Tongji Hospital, Wuhan, China	n.r.	150	age n.r., 32% female	45.3
Salacup G, Bryan K	retrospective cohort	Philadelphia, USA	1 March- 24 April, 2020	244	median age 66 (IQR 58-76), 49% female	21.31
Satici C, Demirkol MA	retrospective cohort	Gaziosmanpasa Research and Training Hospital, University of Health Sciences, Istanbul, Turkey	2 April- 1 May, 2020	681	mean age 56.9±15.7, 49% female	8.08
Shahriarirad R, Khodamoradi Z	retrospective cohort	university affiliated hospitals in Shiraz, Iran	20 February-20 March, 2020	113	mean age 53.7±16.58, 37.2% female	7.96
Violi F, Cangemi R	retrospective cohort	5 COVID-19 dedicated centers in Italy	March-April, 2020	319	age n.r., 39.5% female	20.06
Wang D, Yin Y	retrospective cohort	Zhongnan Hospital of WuhanUniversity and Xishui People’s Hospital, Wuhan, China	until 10 February, 2020	107	median age 51 (IQR 36-65), 46.7% female	17.76
Wang K, Zuo P TRAINING COHORT	prospective cohort	First People’s Hospital of Jiangxia District in Wuhan, China	7 January-11 February, 2020	296	mean age 47.32 ±14.95, 52.7% female	6.42
Wang K, Zuo P VALIDATION COHORT	retrospective cohort	Infection department of Union Hospital in Wuhan, China	1 January-20 February, 2020	44	mean age 55.2±16.8, 45.5% female	31.82
Xu B, Fan CY	retrospective cohort	Hubei Provincial Hospital of traditional Chinese and Western medicine, Wuhan, China	26 December, 2019-1 March, 2020	145	age n.r., 47.6% female	19.31
Yang H, Yang LC	retrospective cohort	Tonji Hospital, Wuhan, China	29 January.20 March, 2020	94	age n.r., 52% female	13.83
Yao Q, Wang P	retrospective cohort	Dabieshan Medical Center, Huanggang city, Hubei Province, China	30 January- 11 February, 2020	108	median age 52 (IQR 37-58), 50.4% female	11.11
Ye W, Chen G	retrospective cohort	Wuhan Pulmonary Hospital, Hubei Province, China	1 January - 16 March, 2020	349	median age 62 (IQR 21-69), 48% female	14.90
Yu C, Lei Q	retrospective cohort	Tongji Hospital, Wuhan, China	14 January- 28 February, 2020	1464	median age 64 (IQR 51-71) 49.7 % female	61.50

Zhang L, Yan X	retrospective cohort	Wuhan Asia General Hospital, Wuhan, China	14 January-28 February, 2020	1464	median age: 64 (IQR 51-71), 49.7% female	14.48
Zhao L, Zhang YP	retrospective cohort	Tongji Hospital, Wuhan, China	9 February-16 February, 2020	51	n.r.	11.74
Zhao X, Wang K	prospective cohort	First People's Hospital of Jiangxia District, Wuhan, China	7 January-28 February, 2020	532	age n.r., 53.8 % female	54.51
Zhou F, Yu T	retrospective cohort	Jinyintan Hospital and Wuhan Pulmonary Hospital, Wuhan, China	29 December, 2019-31 January, 2020	191	mean age 56 (IQR 46-67), 38% female	28.27
**Studies assessing the risk for intensive care requirement in all COVID-19 cases**						
Aggarwal S, Garcia-Telles N	retrospective cohort	Des Moines, Iowa, USA	1 March- 4 April, 2020	16	mean age 67 (IQR: 38-95), 25% female	50.00
Al-Samkari H, Leaf RK	retrospective cohort	5 hospitals in Boston, Massachusetts, USA	1 March-5 April, 2020	400	age n.r., 43% female	36.00
Asghar MS, Kazmi, SJH	retrospective cohort	Karachi, Pakistan	March-April, 2020	100	mean age: 52.58±15.68, 31% female	33.00
Bhargava A, Fukushima EA	retrospective observational study	St John Hospital, Detroit, Michigan, USA	8 March-8 April, 2020	197	mean age: 60.6±16.2, 47.7% female	38.07
Burian E, Jungman F	retrospective cohort	Munich, Germany	March-April, 2020	65	mean age: 61.5±17, 35.4% female	43.08
Cai SH, Liao W	retrospective cohort	Dongguan People's Hospital, Nanfang hospital and the First Affiliated Hospital of Xiamen University, China	23 January-14 February, 2020	96	age n.r., 43.75% female	n.r.
Cecconi M, Piovani D	retrospective cohort	Humanitas Research Hospital, Rozzano, Italy	22 February- 22 March, 2020	239	mean age: 63.9 ± 14.0, 29.3% female	17.15
Chan SSW, Dheepa C	retrospective cohort	Tan Tock Seng Hospital, Singapore	24 February-28 March, 2020	75	median age 50 (IQR: 30-62), 33.3% female	26.67
Chen J, Tangkai Q	retrospective cohort	Shanghai Public Health Clinical Center, Shanghai, China	20 January-6 February, 2020	249	median age:51 (IQR 36–64), 49.4% female	8.84
Chen R, Sang L	retrospective cohort	Wuhan, China	until 22 March, 2020	548	mean age: 56±14.5, 42.9% female	8.76
Cugno M, Meroni PL	prospective ohort	Milan, Italy	n.r.	31	median age: 59 (range 31-85), 32.3% female	45.16
D'Alessandro M, Cameli P	prospective cohort	Siena University Hospital, Italy	n.r.	22	median age: 63 (IQR: 59-68), 27.3% female	54.55
Du RH, Liu LM	retrospective observational study	Wuhan Pulmonary Hospital, Tianyou Hospital and Central Hospital of Wuhan, China	25 December, 2019-15 February, 2020	109	mean age: 70.7±10.9, 32.1% female	46.79
Fan BE, Chong VCL	retrospective cohort	National Centre for Infectious Diseases, Singapore	23 January - 28 February, 2020	67	median age: 42 (IQR: 35-54), 44,8% female	13.43
Feng Y, Ling Y	retrospective cohort	Jinyintan Hospital in Wuhan, Shanghai Public Health Clinical Center in Shanghai, and Tongling People's Hospital in Anhui, China	1 January. 15 February, 2020	476	n.r.	14.71
Galloway JB, Norton S	observational cohort	King's College Hospital and Princess Royal University Hospital, London, UK	1 March- 17 April, 2020	1157	median age: 71 (IQR 57,82), 42.4% female	13.57
Goshua G, Pine AB	cross-sectional study	Yale New Haven Hospital, Connecticut, USA	13 April-24 April, 2020	68	mean age: 62±16, 40% female	70.59
Hong KS, Lee KH	retrospective cohort	Yeungnam University Medical Center in Daegu, South Korea	in December, 2019	98	mean age: 55.4±17.1, 61.2% female	13.27

Huang C, Wang Y	prospective cohort	Jinyintan Hospital, Wuhan, China	16 December, 2019-2 January 2020	41	median age: 49 (IQR: 41-58), 27·0% female	31.71
Ihle-Hansen H, Berge T	n.r.	University of Oslo, Norway	3 March-31 March, 2020	42	median age: 72.5 (range 30-95), 33.3% female	21.23
Israelsen SB, Kristiansen KT	retrospective case-series	Hvidovre Hospital, Copenhagen, Denmark	10 March-23 April, 2020	175	median age:71 (IQR 55-81), 51.4%	15.43
Khamis F, Al-Zakwani I	retrospective case-series	Royal Hospital and Al Nahdha Hospital, Oman	24 February-24 April, 2020	63	mean age: 48±16, 15% female	38.10
Lagi F, Piccica M	retrospective cohort	Infectious and Tropical Disease Unit of the University Hospital, Florence,Tuscany, Italy	5 February-26 March, 2020	84	median age: 62 (IQR 51-72), 34.5% female	19.05
Li H, Xiang X	retrospective cohort	Tianyou Hospital of Wuhan University of Science and Technology, China	18 January-26 February, 2020	132	mean age: 62.05±12.68, 43.2% female	12.12
Liu R, Wang Y	retrospective cohort	Renmin Hospital of Wuhan University, China	22 January-25 February, 2020	154	mean age: 64±14, 45.5% female	28.57
Liu Y, Yang Y	retrospective case-series	Shenzhen Third People’s Hospital, China	10 January-20 January, 2020	12	age n.r., 25% female	50.00
McElvaney OJ, McEvoy NL	n.r.	Royal College of Surgeons in Ireland, Dublin, Ireland	n.r.	40	mean age: 55.5±17.7, 37.5% female	50.00
Murk J, Biggelar R	retrospective cohort	Elisabeth-Tweesteden Hospital, the Netherlands	26 February-20 March, 2020	100	age n.r., 33% female	19.00
Omrani-Nava V, Maleki I	case controll	Mazandaran University of Medical Sciences, Iran	February-March, 2020	93	mean age: 56.3±15.2, 45.2% female	n.r.
Ortiz-Bizuela E, Villanueva-Reza M	prospective cohort	211-bed referral hospital for adults, Mexico City, Mexico	26 February-23 March, 2020	140	median age: 49 (IQR 39-61.25), 39.3% female	20.71
Petrilli CM, Jones SA	prospective cohort	NYU Langone Health, New York, USA	1 March-8April, 2020	2729	median age: 63 (IQR 51.74), 38.7% female	36.28
Romana PF, Fabio DZ	retrospective cohort	Fondazione Policlinico Universitario Agostino Gemelli IRCCS in Rome, Italy	6 March- 16 April, 2020	515	median age: 65 (IQR 53-77), 37.3% female	14.95
Suleyman G, Fadel RA	retrospective case-series	Henry Ford Health System in metropolitan Detroit, Michigan, USA	9 March-17 March, 2020	335	mean age: 61.4±15.4, 53.5% female	42.90
Sun DQ, Wang TY	retrospective cohort	The First Affiliated Hospital of Wenzhou Medical University, China	February, 2020	32	median age: 61 (IQR 54-73),37.5% female	28.13
Urra JM, Cabrera CM	retrospective case-control study	University Hospital of Ciudad Real, Spain	1 March-15 April, 2020	172	age n.r., 28.3% female	15.70
Wang DW, Hu B	retrospective case-series	Zhongnan Hospital, Wuhan, China	1 January- 28 January	138	median age 56 (IQR: 42-68) 45·7 % female	26.09
Wang F, Hou H	retrospective cohort	Tongji Hospital, Wuhan, China	January, 2020	65	mean age: 57.11±13.03, 43% female	23.08
Wang R, Pan M	retrospective cohort	No.2 People’s Hospital of Fuyang City, China	20 January-9 February, 2020	125	mean age: 41.46±15.09, 43.2% female	20.00
Wu J, Huang J	retrospective cohort	Wuhan Hankou Hospital and No. 6 Hospital of Wuhan, China	26 December, 2019- 15 March, 2020	2041	age NA, 58.2% female	34.15
Yang L, Liu J	retrospective case-series	Yichang Central People's Hospital, a designated hospital in Yichang, Hubei Province, China	30 January-8 February, 2020	200	mean age: 55±17.1, 51% female	14.50

Zeng Z, Ma YAC	retrospective cohort	5 hospitals in China	22 January-14 March, 2020	461	median age: 45 (IQR 34.5-57), 51.48 % female	11.93
Zhou Y, Fu B	n.r.	The First Affiliated Hospital of University of Science and Technology, Hefei, Anhui, China	n.r.	33	age n.r., 33.3% female	36.36
**Studies assessing the risk for mortality in critically ill COVID-19 patients**						
Auld S, Caridi-Scheible M	retrospective cohort	6 COVID-19 designated ICU in 3 hospitals in Atlanta, Georgia, USA	6 March-17 April, 2020	217	median age: 64 (IQR: 54-73), 45.2% female	29.66
Bhatraju KP, Ghassemieh BJ	retrospective case-series	9 hospitals in the USA	24 February-March 9, 2020	28	mean age: 64±18, 37% female	42.86
Borobia A, Carcas A	retrospective cohort	La Paz University Hospital, Madrid, Spain	25 February-19 April, 2020	75	median age 64 (IQR 54-71), 24% female	73.33
Cen Y, Chen X	retrospective cohort	Huoshenshan Hospital, General Hospital of the Central Theatre Command of the PLA, and mobile cabin hospitals in Wuhan, China	from 10 January, 2020	65	age n.r., 50.8% female	66.15
Cummings MJ, Darryl Abrams	prospective observational cohort	two NewYork-Presbyterian hospitals affiliated with Columbia University Irving Medical Center in northern Manhattan, USA	2 March-April 1, 2020	1150	median age: 62 (IQR 51–72), 33% female	22.35
Fan H, Zhang L	retrospective cohort	Jinyintan Hospital, Wuhan, China	30 December, 2019-16 Fenruary, 2020	73	mean age: 58.36±14.31, 32.9% female	64.38
He XW, Lai JS	retrospective cohort	Tongji Medical College, Huazhong University of Science and Technology, China	3 February. 24 February, 2020	54	median age: 68 (IQR 59.8-74.39, 37% female	48.15
Huang W, Li C	retrospective cohort	Tongji Hospital, Wuhan, China	29 January-6 March, 2020	615	age n.r., 38.2% female	37.72
Li J, Li M	retrospective cohort	the Central Hospital of Wuhan, China	1 January- 20 February, 2020	134	median age: 67 (IQR 56-75), 38.98 % female	71.19
Xu J, Yang X	retrospective cohort	Wuhan Union Hospital, Jinyintan Hospital, and Wuhan Third Hospital, China	12 January-3 February, 2020	239	mean age: 62.5±13.3, 40.2% female	61.51
Zou X, Li S	retrospective cohort	Tongji Hospital, Wuhan, China	10 January-10 February, 2020	154	mean age: 60.68±13, 56.5% female	33.77

Supplementary Table 1: Characteristics of included studies
In-hospital mortality: all patients were either dead or discharged· and no unclosed cases were included·
ICU=intensive care unit, SD=standard deviation, IQR=interquartile range, n.r.= not reported

Study name	N^0 of patients in the analysis (N^0 of studies)	Weighted Mean Difference with worse prognosis (95% Confidence Interval)	p-value	I-squared test (p-value)
**Mortality in "mixed" population (deceased vs discharged)**				
White blood cell × 10^9/L	7743 (20)	**2.35 (1.96, 2.83)**	**p<0.001**	64.5% (p<0.001)
Lymphocyte × 10^9/L	9780 (17)	**-0.35 (-0.43, -0.27)**	**p<0.001**	94.2% (p<0.001)
CD3+ lymphocyte cell/µL	2775 (4)	**-329.71 (-370.82, -288.59)**	**p<0.001**	60.1% (p=0.057)
CD4+ lymphocyte cell/µL	2775 (4)	**-164.24 (-190.51, -137.97)**	**p<0.001**	67.0% (p=0.028)
CD8+ lymphocyte cell/µL	2775 (4)	**-115.45 (-130.61, -100.30)**	**p<0.001**	55.7% (p=0.080)
Neutrophil granulocyte × 10^9/L	7210 (12)	**2.67 (2.12, 3.21)**	**p<0.001**	71.7% (p<0.001)
Eosinophil granulocyte × 10^9/L	762 (3)	**-0.02 (-0.03, -0.01)**	**p=0.003**	74.6% (p=0.019)
Monocyte × 10^9/L	2670 (7)	**-0.05 (-0.08, -0.03)**	**p<0.001**	0.0% (p=0.583)
Platelet × 10^9/L	9570 (20)	**-25.66 (–35.56, -15.76)**	**p<0.001**	81.8% (p<0.001)
Haemoglobin g/L	5522 (14)	**-3.69 (-6.51, -0.87)**	**p=0.010**	71.9% (p<0.001
C-reactive protein mg/L	9093 (21)	**65.65 (43.79, 87.50)**	**p<0.001**	99.4% (p<0.001)
Lactate dehydrogenase (U/L)	8314 (16)	**203.79 (151.86, 255.71)**	**p<0.001**	95.2% (p<0.001)
Procalcitonin ng/mL	9900 (12)	**0.38 (0.30, 0.47)**	**p<0.001**	91.8% (p<0.001)
Fibrinogen g/L	6476 (7)	**0.32 (0.13, 0.50)**	**p=0.001**	52.1% (p=0.051)
D-dimer mg/L	12540 (22)	**1.31 (1.05, 1.57)**	**p<0.001**	84.5% (p<0.001)
Ferritin µg/L	8274 (11)	**550.20 (347.97, 752.43)**	**p<0.001**	15.8% (p=0.305)
Creatine kinase (U/L)	5047 (9)	**77.59 (55.31, 99.86)**	**p<0.001**	81.4% (p<0.001)
Interleukin-1 pg/mL	1116 (3)	0.27 (-0.14, 0.67)	p=0.197	95.1% (p<0.001)
Interleukin-6 pg/mL	7023 (8)	**84.26 (49.23, 119.30)**	**p<0.001**	97.5% (p<0.001)
**Mortality among critically ill patients (deceased vs discharged)**				
White blood cell × 10^9/L	326 (3)	-0.27 (-1.64, 1.10)	p=0.697	19.9% (p=0.287)
Lymphocyte × 10^9/L	403 (4)	-0.12 (-0.28, 0.03)	p=0.119	75.5% (p=0.007)
Platelet × 10^9/L	401 (4)	**-30.19 (-44.88, -15.50)**	**p<0.001**	0.0% (p=0.896)
C-reactive protein mg/L	423 (4)	**45.36 (23.50, 67.21)**	**p<0.001**	35.3% (p=0.200)
Lactate dehydrogenase (U/L)	189 (3)	**129.34 (67.73, 190.94)**	**p<0.001**	34.1% (p=0.219)
Procalcitonin ng/mL	124 (3)	0.13 (-0.23, 0.48)	p=0.479	88.9% (p<0.001)
D-dimer mg/L	411 (4)	1.69 (-0.61, 3.99)	p=0.149	85.5% (p<0.001)
**Intensive care requirement (ICU vs non-ICU)**				
White blood cell × 10^9/L	5130 (22)	**1.53 (1.04, 2.02)**	**p<0.001**	68.8% (p<0.001)
Lymphocyte × 10^9/L	8063 (23)	**-0.30 (-0.37, -0.23)**	**p<0.001**	87.0% (p<0.001)
CD3+ lymphocyte cell/µL	269 (3)	**-322.56 (-589, -55.54)**	**p=0.018**	83.5% (p=0.002)
CD4+ lymphocyte cell/µL	302 (4)	**-142.98 (-242.12, -43.85)**	**p=0.005**	82.2% (p=0.001)
CD8+ lymphocyte cell/µL	302 (4)	**-186.52 (-254.84, -118.21)**	**p<0.001**	74.3% (p=0.009)
Neutrophil × 10^9/L	2357 (18)	**2.47 (1.71, 3.23)**	**p=0.037**	75.2% (p<0.001)
Monocyte × 10^9/L	510 (6)	-0.06 (-0.14, 0.02)	p=0.146	58.7% (p=0.033)
Platelet × 10^9/L	2606 (21)	-4.26 (–18.44, 8.87)	p=0.492	66.4% (p<0.001)
Haemoglobin g/L	1647 (14)	**-7.39 (-11.65, -3.14)**	**p=0.001**	64.1% (p=0.001)
C-reactive protein mg/L	4402 (17)	**68.51 (53.19, 83.83)**	**p<0.001**	79.8% (p<0.001)
Lactate dehydrogenase (U/L)	2425 (16)	**190.91 (129.40, 252.42)**	**p<0.001**	90.4% (p<0.001)
Procalcitonin ng/mL	3763 (8)	**0.21 (0.05, 0.37)**	**p=0.008**	95.6% (p<0.001)
Fibrinogen g/L	695 (3)	**1.04 (0.66,1.43)**	**p<0.001**	0.0% (p=0.900)
D-dimer mg/L	3417 (15)	**0.77 (0.50, 1.04)**	**p=0.007**	81.1% (p<0.001)
Ferritin µg/L	2168 (3)	**328.28 (181.58, 474.99)**	**p<0.001**	15.8% (p=0.305)
Creatine kinase (U/L)	1586 (8)	**54.07 (28.37, 79.77)**	**p<0.001**	35.2% (p=0.148)
Interleukin-6 pg/mL	258 (4)	**26.67 (15.98, 37.35)**	**p<0.001**	0.0% (p=0.592)

Supplementary Table 2: Summary for the results of the quantitative synthesis for continuous outcomes.

Laboratory parameter	Threshold	N^0 of patients in the analysis (N^0 of studies)	Odds ratio with worse prognosis (95% Confidence Interval)	p-value	I-squared test (p-value)
**Mortality in "mixed" population (deceased vs discharged)**					
White blood cell × 10^9/L	<3.5	191 (2)	0.98 (0.24, 4.04)	p=0.976	0.0% (p=0.829)
	<4.0	4609 (7)	**0.38 (0.20, 0.72)**	**p=0.003**	40.6% (p=0.120)
	>9.5	302 (3)	**3.70 (1.72, 7.69)**	**p=0.001**	0.0% (p=0.523)
	>10.0	4747 (7)	**6.25 (2.86, 14.29)**	**p<0.001**	85.2 (p<0.001)
	>11.0	96 (1)	**6.67 (2.44, 20.0)**	**p<0.001**	-
Lymphocyte × 10^9/L	<0.5	28 (1)	14.67 (0.55, 449.11)	p=0.108	-
	<0.8	723 (5)	**3.74 (1.77, 7.92)**	**p=0.001**	65.5% (p=0.021)
	<1.0	28 (1)	0.32 (0.03, 3.38)	p=0.347	-
	<1.1	2107 (4)	1.79 (0.41, 7.88)	p=0.442	88.4% (p<0.001)
	<1.5	1341 (3)	2.18 (0.28, 16.76)	p=0.456	71.8% (p=0.029)
Platelet × 10^9/L	<100	328 (3)	3.42 (0.40, 29.38)	p=0.262	63.7% (p=0.064)
	<125	630 (3)	**8.10 (3.54, 18.54)**	**p<0.001**	32.7% (p=0.227)
	<150	1644 (5)	1.07 (0.66, 1.74)	p=0.770	0.0% (p=0.680)
	>400	204 (2)	3.37 (0.12, 91.10)	p=0.471	70.5% (p=0.066)
	>450	113 (1)	1.06 (0.12, 9.26)	p=0.960	-
C-reactive protein mg/L	>3.0	102 (1)	7.15 (0.41, 125.74)	p=0.179	-
	>5.0	528 (2)	6.25 (0.07, 592.58)	p=0.430	77.2 (p=0.036)
	>8.0	146 (2)	0.41 (0.11, 1.58)	p=0.195	0.0% (p=0.452)
	>10.0	1823 (4)	**4.84 (1.49, 15.67)**	**p=0.009**	45.8% (p=0.137)
	>50.0	375 (3)	1.34 (0.36, 5.02)	p=0.667	48.3% (p=0.145)
	>100	514 (3)	**2.49 (1.42, 4.35)**	**p=0.001**	14.7% (p=0.310)
	>150	1001 (2)	**2.92 (2.22, 3.84)**	**p<0.001**	0.0% (p=0.826)
Lactate dehydrogenase (U/L)	>214	3014 (2)	2.74 (0.14, 53.68)	p=0.506	77.1% (p=0.036)
	>245	141 (1)	**22.59 (2.96, 172.16)**	**p=0.003**	-
	>250	763 (3)	**10.88 (4.48, 26.39)**	**p<0.001**	0.0% (p=0.705)
	>350	27 (1)	0.07 (0.001, 1.91)	p=0.114	-
	>440	1492 (2)	1.56 (0.48, 5.13)	p=0.460	32.1% (p=0.225)
	>445	561 (2)	2.59 (0.12, 57.11)	p=0.548	81.7% (p=0.019)
Procalcitonin ng/mL	>0.05	4167 (3)	10.38 (0.26, 411.70)	p=0.213	96.0% (p<0.001)
	>0.10	164 (1)	**9.09 (4.17, 20.00)**	**p<0.001**	-
	>0.25	164 (1)	**12.50 (3.85, 33.33)**	**p<0.001**	-
	>0.50	1392 (4)	**11.97 (4.75, 30.16)**	**p<0.001**	59.4% (p=0.061)
D-dimer mg/L	>0.50	2920 (8)	**4.30 (1.55, 11.98)**	**p=0.005**	83.7% (p<0.001)
	>0.55	77 (1)	**9.77 (3.05, 31.33)**	**p<0.001**	-
	>1.0	895 (6)	**6.63 (3.62, 12.14)**	**p<0.001**	45.1% (p=0.105)
	>1.11	85 (1)	**4.07 (142, 11.67)**	**p=0.009**	-
	>2.0	1983 (2)	6.82 (0.77, 60.36)	p=0.084	66.1% (p=0.086)
	>2.5	280 (2)	8.77 (0.28, 270.16)	p=0.214	78.8% (p=0.030)
	>3.0	116 (2)	**18.09 (4.63, 70.69)**	**p<0.001**	0.0% (p=0.330)
Creatine kinase (U/L)	>185	428 (3)	**3.14 (1.87, 5.27)**	**p<0.001**	0.0% (p=0.458)
	>190	135 (2)	1.48 (0.47, 4.68)	p=0.506	0.0% (p=0.774)
**Intensive care requirement (ICU vs non-ICU)**					
White blood cell × 10^9/L	<3.5	460 (4)	**0.42 (0.18, 0.96)**	**p=0.039**	0.0% (p=0.501)
	<4.0	963 (7)	0.71 (0.37, 1.39)	p=0.323	32.9% (p=0.177)
	>9.5	482 (5)	**4.53 (1.95, 10.52)**	**p<0.001**	26.8% (p=0.243)

	>10.0	725 (4)	**2.64 (1.22, 5.71)**	**p=0.014**	61.3% (p=0.051)
	>11.0	96 (1)	**5.67 (2.21, 14.59)**	**p<0.001**	-
Lymphocyte × 10^9/L	<0.4	100 (1)	0.59 (0.07, 5.08)	p=0.629	-
	<0.6	100 (1)	1.08 (0.32, 3.95)	p=0.899	-
	<0.8	100 (1)	1.39 (0.49, 3.95)	p=0.542	-
	<1.0	831 (5)	**4.54 (2.58, 7.95)**	**p<0.001**	22.3% (p=0.273)
	<1.1	1267 (8)	**2.64 (1.49, 4.70)**	**p=0.001**	36.4% (p=0.138)
	<1.5	100 (1)	1.30 (0.47, 3.66)	p=0.613	-
	>3.2	315 (4)	1.38 (0.29, 6.67)	p=0.689	0.0% (p=0.687)
Neutrophil granulocyte × 10^9/L	>6.3	186 (3)	**2.32 (1.23, 4.37)**	**p=0.009**	0.0% (p=0.416)
	<1.8	109 (1)	0.12 (0.01, 2.24)	p=0.154	-
	<1.0	67 (1)	**439.40 (19.09, 9658.21)**	**p<0.001**	-
Platelet × 10^9/L	<100	331 (5)	1.60 (0.61, 4.19)	p=0.335	28.3% (p=0.233)
	<125	926 (5)	1.39 (0.80, 2.42)	p=0.243	0.0% (p=0.755)
	<150	479 (3)	1.05 (0.67, 1.65)	p=0.840	0.0% (0.641)
	>350	132 (1)	0.34 (0.02, 6.17)	p=0.468	-
	>400	158 (2)	**3.63 (1.13, 11.68)**	**p=0.031**	0.0% (p=0.347)
C-reactive protein mg/L	>5.0	499 (1)	16.00 (0.97, 263.34)	p=0.052	-
	>6.0	71 (1)	0.40 (0.12, 1.36)	p=0.143	-
	>10.0	948 (6)	**3.85 (1.21, 12.22)**	**p=0.022**	55.4% (p=0.047)
	>50.0	108 (2)	**5.53 (1.45, 21.15)**	**p=0.012**	0.0% (p=0.625)
	>100	730 (2)	**6.25 (4.23, 9.23)**	**p<0.001**	0.0% (p=0.850)
Lactate dehydrogenase (U/L)	>240	12 (1)	0.28 (0.01, 8.42)	p=0.465	-
	>245	40 (1)	7.06 (0.79, 62.72)	p=0.080	-
	>248	52 (1)	6.60 (0.77, 56.37)	p=0.085	-
	>250	301 (3)	**9.44 (4.12, 24.02)**	**p<0.001**	0.0% (p=0.953)
	>550	67 (1)	**8.48 (1.71, 42.13)**	**p=0.009**	-
Procalcitonin ng/mL	>0.05	517 (4)	14.78 (6.06, 36.03)	p<0.001	48.8% (p=0.118)
	>0.10	39 (1)	3.50 (0.82, 14.93)	p=0.090	-
	>0.12	132 (1)	3.12 (0.73, 13.23)	p=0.124	-
	>0.25	40 (1)	4.33 (0.62, 30.25)	p=0.139	-
	>0.50	1389 (7)	1.92 (0.92, 4.00)	p=0.081	57.6% (0.92, 4.00)
D-dimer mg/L	>0.50	837 (5)	**3.37 (1.90, 5.95)**	**p<0.001**	0.0% (p=0.780)
	>0.55	54 (1)	**6.58 (1.81, 23.96)**	**p=0.004**	-
	>1.00	400 (1)	**2.70 (1.75, 4.17)**	**p<0.001**	-
	>2.50	400 (1)	1.26 (0.69, 2.32)	p=0.454	-

Supplementary Table 3: Summary for the results of the quantitative synthesis for on admission laboratory thresholds

Study authors and year of publication	Results of the study regarding the association between baseline laboratory parameter and mortality/intensive care requirement
**Studies assessing the risk for mortality in all COVID-19 patients**	
Chen X, Zhao B 2020	Interleukin-6 <100 pg/mL vs ≥100 pg/mL (0/42 vs 3/3 death, respectively; p=0.001)<br>*(Comment from review authors: This study was excluded from the quantitative synthesis because of the possibility of overlapping with other studies with higher patient number. See "Methods" section of the manuscript.)*
Galloway JB, Norton S 2020	Absolute lymphocyte count x10^9/L HR=0.46 (95% CI: 0.26, 0.84), p=0.010<br>Absolute neutrophil count x10^9/L HR=1.06 (95% CI: 1.02, 1.09), p<0.001<br>C-reactive protein mg/L HR= 1.06 (95% CI: 1.02, 1.09), p<0.001<br>*(Comment from review authors: HRs were adjusted for age and sex.)*
Li L, Yang L 2020	Total white blood cell count (p=0.201)<br>(survivor: 4.6x10^9/L (3.8–5.8); non-survivor 5.2x10^9/L (3.9–5.9))<br>Absolute lymphocyte count (p=0.001)<br>(survivor: 1.2x10^9/L (0.9–1.6); non-survivor 0.8x10^9/L (0.6–1.2))<br>Absolute neutrophil count (p=0.045)<br>(survivor: 2.8x10^9/L (2.2–3.6); non-survivor 3.8x10^9/L (2.7–5.2))<br>Platelet count (p=0.002)<br>(survivor: 181x10^9/L (147–224); non-survivor 136x10^9/L (112–173))<br>Haemoglobin (p=0.717)<br>(survivor: 131 g/L (120–146); non-survivor 133 g/L (16.8))<br>Lactate dehydrogenase (p<0.001)<br>(survivor: 204 U/L (173–248); non-survivor 373 U/L (151))<br>Creatine kinase<br>(survivor: 59.5 U/L (40.8–116); non-survivor 186 U/L (124–300))<br>C-reactive protein (p<0.001)<br>(survivor: 7.7 mg/L (3.9–15.7); non-survivor 77 mg/L (44))<br>D-dimer (p=0.064)<br>(survivor: 0.3 mg/L (0.2–0.5); non-survivor 0.6 mg/L (0.3–2.1))<br>Ferritin (p=0.094)<br>(survivor: 489 μg/L (381); non-survivor 810 μg/L (409))<br>*(Comment from review authors: Values are given in mean (SD) or median (IQR). Haemoglobin, lactate dehydrogenase, and C-reactive protein levels were reported in different measures (median and mean) in the two group. This study was excluded from the quantitative synthesis because of the possibility of overlapping with other studies with higher patient number. See "Methods" section of the manuscript.)*
Li Y, Peng S 2020	Absolute lymphocyte count <1.1x10^9/L<br>(among survivors 18/20 vs among non-survivors 4/5; p=0.504)<br>Total white blood cell count <4x10^9/L<br>(among survivors 11/20 vs among non-survivors 1/5; p=1.000)<br>Total white blood cell count <9.5x10^9/L<br>(among survivors 9/20 vs among non-survivors 4/5; p=0.322)<br>Increase of LDH<br>(among survivors 11/20 vs among non-survivors 3/5; p=1.000)<br>Increase of C-reactive protein<br>(among survivors 13/20 vs among non-survivors 3/5; p=1.000)<br>Increase of ferritin<br>(among survivors 7/20 vs among non-survivors 2/5; p=1.000)<br>Increase of D-dimer<br>(among survivors 9/20 vs among non-survivors 2/5; p=1.000)<br>*(Comment from review authors: Thresholds were not specified for lactate dehydrogenase, C-reactive protein, ferritin, and D-dimer.)*
Liu Y, Sun W 2020	Platelet count <138 x10^9/L HR=5.42 (95% CI: 1.89, 15.60) → first quartile<br>Platelet count 138–174 x10^9/L HR=2.20 (95% CI: 0.69, 7.02) → second quartile

	Platelet count 174–213 x10^9/L HR=2.29 (95% CI: 0.72, 7.31) → third quartile<br>Platelet count >213x10^9/L HR=0.46 (95% CI: 0.26, 0.84) → fourth quartile<br>P value trend: <0.001 (estimated using median value of each quartile)<br>*(Comment from review authors: only the first threshold provided significant results.)*
Omrani-Nava V, Maleki I 2020	Lymphopenia OR=7.86 (95% CI: 0.43, 142.74), p=0.163<br>Thrombocytopenia OR=0.53 (95% CI: 0.04, 6.67), p=0.624<br>CRP (positive) OR=0.56 (95% CI 0.08, 3.75), p=0.553<br>*(Comment from review authors: data from 93 confirmed COVID-19 patients and 186 healthy controls Normal values reported: absolute lymphocyte count: 1,000-4,000 per mm^3; platelet: 150,000-450,000 per mm^3)*
Price-Haywood EG, Burton J 2020	Absolute lymphocyte count <1000/µL HR=1.33 (95% CI: 1.01, 1.74)<br>Platelet count <150,000/µL HR=1.26 (95% CI: 1.00, 1.60)<br>Procalcitonin >0.25 ng/mL HR=1.40 (95% CI: 1.06, 1.84)<br>C-reactive protein >8.2 ng/mL HR=1.01 (95% CI: 0.49, 2.08)<br>*(Comment from review authors: HRs were adjusted for race, age, sex, Charlson Comorbidity Index score, indicators for baseline vital signs and laboratory measures above or below predefined clinical thresholds (respiratory rate; levels of aspartate aminotransferase, venous lactate, creatinine, bilirubin, procalcitonin, and C-reactive protein; and counts of lymphocytes and platelets).*
Rivera-Izquierdo M, Valero-Ubierna MDC 2020	Lymphocytes HR=1.00 (95% CI: 0.99, 1.00)<br>Neutrophils HR=1.00 (95% CI: 0.99, 1.01)<br>Haemoglobin HR=1.00 (95% CI: 0.88, 1.13)<br>D-Dimer HR=1.00 (95% CI: 0.99, 1.00)<br>Ferritin HR=1.00 (95% CI: 1.00, 1.00)<br>C-reactive protein HR=1.00 (95% CI: 1.00, 1.00)<br>Procalcitonin HR=1.04 (95% CI: 1.00, 1.08)<br>*(Comment from review authors: HRs were adjusted for age expressed as increments in the hazard of death per unit increase in the variable. However, these units were not reported.)*
Zhang L, Yan X 2020	Total white blood cell count C-index=0.625 (95% CI: 0.571, 0.676)<br>Absolute lymphocyte count C-index=0.872 (95% CI: 0.832, 0.906)<br>Absolute neutrophil count C-index=0.773 (95% CI: 0.725, 0.817)<br>Platelet count C-index=0.781 (95% CI: 0.734, 0.824)<br>Haemoglobin C-index=0.583 (95% CI: 0.528, 0.635)<br>D-dimer C-index=0.883 (95% CI: 0.842, 0.916)<br>*(Comment from review authors: Similarly to the AUC, C-index=1 corresponds to the best model prediction, and C-index=0.5 represents a random prediction. Source: https://square.github.io/pysurvival/metrics/c_index.html; Accessed 30/08/2020)*
**Studies assessing the risk for intensive care requirement in all COVID-19 patients**	
Bhargava A, Fukushima EA 2020	Leukopenia OR=0.81 (95% CI: 0.31, 2.12), p=0.67<br>Lymphopenia OR=1.47 (95% CI: 0.82, 2.64), p=0.20<br>Thrombocytopenia OR=1.17 (95% CI: 0.56, 2.42), p=0.68<br>Elevated C-reactive protein OR=4.20 (95% CI: 0.51, 34.94), p=0.15<br>Elevated procalcitonin OR=4.29 (95% CI: 1.41, 12.99), p=0.006<br>*(Comment from review authors: Thresholds were not specified)*
Cai SH, Liao W 2020	Absolute lymphocyte count OR=0.684 (95% CI: 0.350, 1.338), p=0.267<br>Absolute neutrophil count OR=0.979 (95% CI: 0.725, 1.322), p=0.889<br>Platelet count OR=0.997 (95% CI: 0.990, 1.004), p=0.398<br>Haemoglobin OR=1.006 (95% CI: 0.981, 1.032), p=0.630<br>Lactate dehydrogenase OR=1.001 (95% CI: 0.994, 1.008), p=0.756<br>Creatine kinase OR=1.002 (95% CI: 1.000, 1.005), p=0.097

	(Comment from review authors: Thresholds were not specified. Data of 96 confirmed COVID-19 cases.)
Cecconi M, Piovani D 2020	Procalcitonin ≥0.5 ng/mL HR=2.86 (95% CI: 1.74, 4.69), p<0.001<br>Interleukin-6 ≥200 pg/mL HR=1.31 (95% CI: 1.00, 1.73), p=0.049<br>Ferritin ≥ 336.2 ng/mL HR=2.49 (95% CI: 1.23, 5.04), p=0.012<br>C-reactive protein ≥5 mg/dL HR=3.63 (95% CI: 1.90, 6.92), p=0.010<br>*(Comment from review authors: Univariable Cox PH Model)*
Chen J, Tangkai Q 2020	Total white blood cell count x10^9/L OR=1.28 (95% CI: 1.08, 1.52), p=0.004<br>Absolute lymphocyte count x10^9/L OR=0.24 (95% CI: 0.08, 0.75), p=0.010<br>CD4+ lymphocyte count per 100 cells/µL OR=0.45 (95% CI: 0.31, 0.64), p<0.001<br>C-reactive protein mg/L OR=1.04 (95% CI: 1.02, 1.05), p=0.67<br>Lactate dehydrogenase (U/L) OR=1.01 (95% CI: 1.00, 1.02), p<0.001<br>*(Comment from review authors: Univariate logistic regression referring to increase or decrease of risk for mortality by each unit of the given parameters)*
Galloway JB, Norton S 2020	Absolute lymphocyte count x10^9/L HR=0.59 (95% CI: 0.30, 1.13), p=0.113<br>Absolute neutrophil count x10^9/L HR=1.09 (95% CI: 1.05, 1.13), p<0.001<br>C-reactive protein mg/L HR= 1.05 (95% CI: 1.03, 1.06), p<0.001<br>(Comment by review authors: HRs were adjusted for age and sex.)
Omrani-Nava V, Maleki I 2020	Lymphopenia OR=1.48 (95% CI: 0.23, 9.51), p=0.676<br>Thrombocytopenia OR=1.79 (95% CI: 0.12, 25.65), p=0.667<br>CRP (positive) OR=2.83 (95% CI 0.48, 16.54), p=0.245<br>*(Comment from review authors: data from 93 confirmed COVID-19 patients and 186 healthy controls.)*
**Studies assessing the risk for mortality among critically ill COVID-19 patients**	
Cummings MJ, Darryl Abrams 2020	Interleukin-6 pg/mL HR=1.11 (95% CI: 1.02, 1.20) (per decile increase)<br>D-dimer µg/mL HR=1.10 (95% CI: 1.01, 1.19) (per decile increase)<br>*(Comment from review authors: HRs were adjusted to initial severity of the disease.)*
Li J, Li M 2020	Platelet count OR=0.998 (95% CI: 0.978, 0.999), p=0.012<br>D-dimer OR=1.112 (95% CI: 0.951, 1.301), p=0.185<br>Lactate dehydrogenase OR=1.004 (95% CI: 1.000, 1.008), p=0.073<br>*Comment from review authors: ORs were adjusted for age, and cardiovascular disease acute respiratory distress syndrome)*

Supplementary Table 4: Results of studies included in the qualitative synthesis
CI: confidence interval; HR: hazard ratio; IQR: interquartile range OR: odds ratio, SD: standard deviation

A

n.a. Not applicable
+ Low risk
? Moderate risk
— High risk

Study	Study participation	Study attrition	Prognostic factor measurement	Outcome measurement	Study confounding [1]	Statistical analysis reporting [2]	Overall risk of bias	Included in meta-analyses
Asghar MS, Kazmi, SJH	+	n.a.	+	+	-	+	-	Yes
Al-Samkari H, Leaf RK	+	n.a.	+	+	-	+	-	Yes
Barman HA, Atici A	+	n.a.	+	+	?	+	+	Yes
Bazzan M, Montaruli B	+	?	+	+	-	+	-	Yes
Bonetti G, Manelli F	+	n.a.	+	+	?	+	+	Yes
Borobia A, Carcas A	+	n.a.	+	+	-	+	-	Yes
Cao J, Tu WJ	+	n.a.	+	+	-	+	-	Yes
Chen L, Yu J	+	n.a.	+	+	?	+	+	Yes
Chen R, Liang W	+	n.a.	+	+	?	+	+	Yes
Chen R, Sang L	+	n.a.	+	+	?	+	+	Yes
Chen X, Zhao B	+	n.a.	+	+	-	+	-	No
Ciceri F, Castagna A	+	n.a.	+	+	?	+	+	Yes
De Biasi S, Meschiari M	+	n.a.	+	+	?	+	+	Yes
Fan JL, Wang H	+	n.a.	+	+	?	+	+	Yes
Galloway JB, Norton S	+	n.a.	+	+	?	?	?	No
Gan J, Li J	+	n.a.	+	+	+	+	+	Yes
Giacomelli A, Ridolfo AL	+	+	+	+	-	+	-	Yes
Javanian M, Bayani M	+	n.a.	+	+	?	+	+	Yes
Li D, Chen Y	+	n.a.	+	+	-	+	-	Yes
Li K, Chen D	+	n.a.	+	+	?	+	+	Yes
Li L, Yang L	+	n.a.	+	+	?	+	+	No
Li Q, Cao Y	+	n.a.	+	+	-	+	-	Yes
Li Y, Peng S	+	n.a.	?	+	?	+	?	No
Liu Y, Sun W	+	n.a	+	+	?	?	?	No

Study	Study participation	Study attrition	Prognostic factor measurement	Outcome measurement	Study confounding [1]	Statistical analysis reporting [2]	Overall risk of bias	Included in meta-analyses
Long H, Nie L	+	n.a.	+	+	-	+	-	Yes
Luo M, Liu J	+	n.a.	+	+	?	+	+	Yes
Mikami T, Miyashita H	+	n.a.	+	+	?	+	+	Yes
Omrani-Nava V, Maleki I	+	n.a.	+	+	-	?	-	No
Price-Haywood EG, Burton J	+	n.a.	+	+	?	?	?	No
Rivera-Izquierdo M, Valero-Ubierna MDC	+	n.a.	+	+	?	?	?	No
Ruan Q, Yang K	+	n.a.	?	+	-	+	-	Yes
Salacup G, Bryan K	+	n.a.	+	+	+	+	+	Yes
Satici C, Demirkol MA	+	n.a.	+	+	+	+	+	Yes
Shahriarirad R, Khodamoradi Z	+	n.a.	?	+	?	+	?	Yes
Violi F, Cangemi R	+	n.a.	+	+	?	+	+	Yes
Wang D, Yin Y	+	n.a.	+	+	-	+	-	Yes
Wang K, Zuo P (training cohort)	+	+	+	+	?	+	+	Yes
Wang K, Zuo P (validation cohort)	+	n.a.	+	+	?	+	+	Yes
Xu B, Fan CY	+	n.a.	+	+	?	+	+	Yes
Yang H, Yang LC	+	n.a.	+	+	?	+	+	Yes
Yao Q, Wang P	+	n.a.	+	+	?	+	+	Yes
Ye W, Chen G	+	n.a.	+	+	+	+	+	Yes
Yu C, Lei Q	+	n.a.	+	+	+	+	+	Yes
Zhang L, Yan X	+	n.a.	+	+	?	?	?	No
Zhao L, Zhang YP	+	n.a.	+	+	-	+	-	Yes
Zhao X, Wang K	+	+	+	+	?	+	+	Yes
Zhou F, Yu T	+	n.a.	+	+	?	+	+	Yes

B

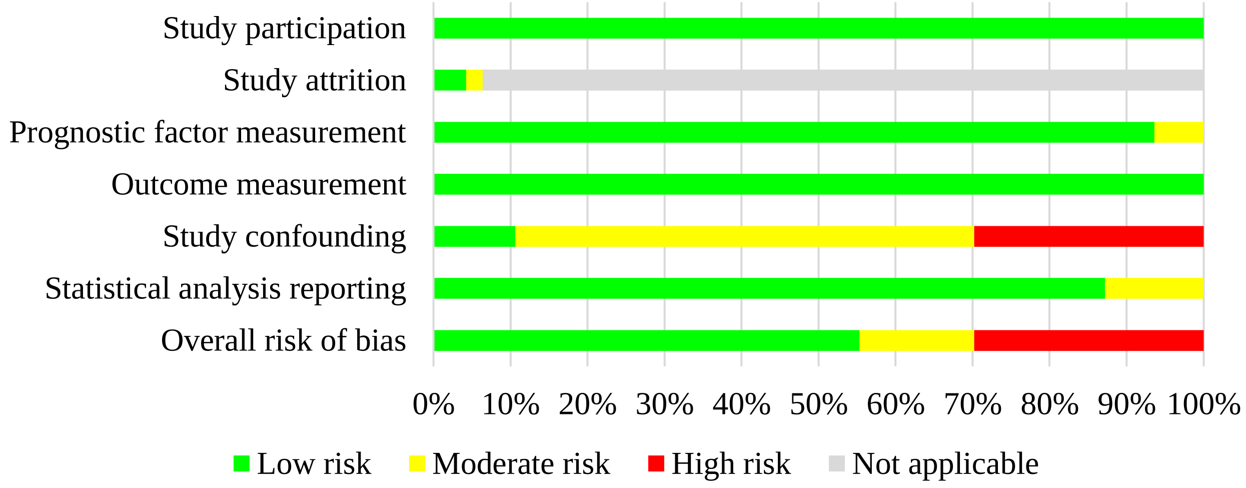

Supplementary Figure 1: Risk of bias assessment on study level [A] and across studies [B] deceased and discharged patients with COVID-19

1: Assessed confounding factors are age, hypertension, heart failure and diabetes 2: As we analyzed raw data in the meta-analyses, statistical approaches of individual studies do no imply risk for this domain

A

n.a. Not applicable
+ Low risk
? Moderate risk
— High risk

Study	Study participation	Study attrition	Prognostic factor measurement	Outcome measurement	Study confounding [1]	Statistical analysis reporting [2]	Overall risk of bias	Included in meta-analyses
Aggarwal S, Garcia-Telles N	+	n.a.	+	?	+	+	+	Yes
Al-Samkari H, Leaf RK	+	n.a.	+	?	+	+	+	Yes
Asghar MS, Kazmi, SJH	+	n.a.	+	?	?	+	?	Yes
Bhargava A, Fukushima EA	+	n.a.	+	?	?	?	-	No
Burian E, Jungman F	+	n.a.	+	?	?	+	?	Yes
Cai SH, Liao W	+	n.a.	+	?	-	?	-	No
Cecconi M, Piovani D	+	n.a.	+	?	?	?	-	No
Chan SSW, Dheepa C	+	n.a.	+	?	-	+	-	Yes
Chen J, Tangkai Q	+	n.a.	+	?	?	?	-	No
Chen R, Sang L	+	n.a.	+	?	+	+	+	Yes
Cugno M, Meroni PL	+	n.a.	+	?	-	+	-	Yes
D'Alessandro M, Cameli P	+	n.a.	+	?	-	+	-	Yes
Du RH, Liu LM	+	n.a.	+	?	+	+	+	Yes
Fan BE, Chong VCL	+	n.a.	+	?	-	+	-	Yes
Feng Y, Ling Y	+	n.a.	+	?	+	+	+	Yes
Galloway JB, Norton S	+	n.a.	+	?	?	?	-	No
Goshua G, Pine AB	+	n.a.	+	?	+	+	+	Yes
Hong KS, Lee KH	+	n.a.	+	?	+	+	+	Yes
Huang C, Wang Y	+	+	+	?	+	+	+	Yes
Ihle-Hansen H, Berge T	+	n.a.	+	?	?	+	?	Yes
Israelsen SB, Kristiansen KT	+	n.a.	+	?	+	+	+	Yes
Khamis F, Al-Zakwani I	+	n.a.	+	?	+	+	+	Yes
Lagi F, Piccica M	+	n.a.	+	?	?	+	?	Yes
Li H, Xiang X	+	n.a.	+	?	-	+	-	Yes
Liu R, Wang Y	+	n.a.	+	?	+	+	+	Yes
Liu Y, Yang Y	+	n.a.	+	?	?	+	?	Yes
McElvaney OJ, McEvoy NL	+	n.a.	+	?	+	+	+	Yes
Murk J, Biggelar R	+	n.a.	+	?	+	+	+	Yes
Omrani-Nava V, Maleki I	+	n.a.	+	?	?	?	-	No
Ortiz-Bizuela E, Villanueva-Reza M	+	+	+	?	+	+	+	Yes
Petrilli CM, Jones SA	+	+	+	?	+	+	+	Yes
Romana PF, Fabio DZ	+	n.a.	+	?	?	+	?	Yes
Suleyman G, Fadel RA	+	n.a.	+	?	+	+	+	Yes
Sun DQ, Wang TY	+	n.a.	+	?	+	+	+	Yes
Urra JM, Cabrera CM	+	n.a.	+	?	+	+	+	Yes
Wang DW, Hu B	+	n.a.	+	?	-	+	-	Yes
Wang F, Hou H	+	n.a.	++	?	-	+	-	Yes
Wang R, Pan M	+	n.a.	+	?	-	+	-	Yes
Wu J, Huang J	+	n.a.	+	?	+	+	+	Yes
Yang L, Liu J	+	n.a.	+	?	-	+	-	Yes
Zeng Z, Ma YAC	+	n.a.	+	?	-	+	-	Yes
Zhou Y, Fu B	+	n.a.	+	?	-	+	-	Yes

B

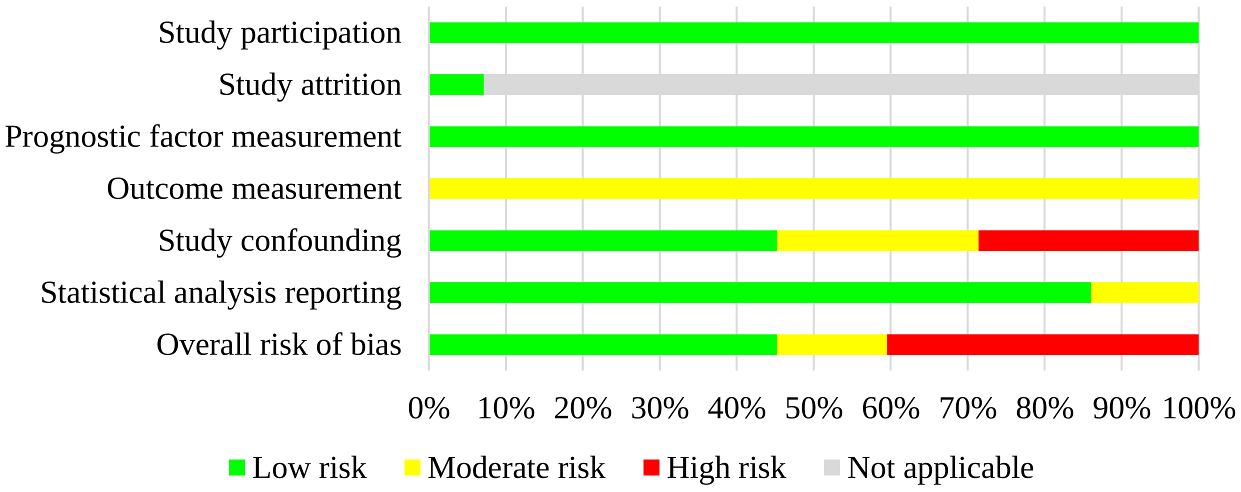

Supplementary Figure 2: Risk of bias assessment on study level [A] and across studies [B] comparing patients with and without intensive care requirement

1: Assessed confounding factors are age, hypertension, heart failure and diabetes 2: As we analyzed raw data in the meta-analyses, statistical approaches of individual studies do no imply risk for this domain

A

n.a. Not applicable
\+ Low risk
? Moderate risk
— High risk

	Study participation	Study attrition	Prognostic factor measurement	Outcome measurement	Study confounding [1]	Statistical analysis reporting [2]	Overall risk of bias	Included in meta-analyses
Auld S, Caridi-Scheible M	?	n.a.	+	+	?	+	?	Yes
Bhatraju KP, Ghassemieh BJ	?	n.a.	+	+	?	+	?	Yes
Borobia A, Carcas A	?	n.a.	+	+	-	+	-	Yes
Cen Y, Chen X	?	n.a.	+	+	?	+	?	Yes
Cummings MJ, Darryl Abrams	?	+	+	+	?	?	-	No
Fan H, Zhang L	?	n.a.	+	+	-	+	-	Yes
He XW, Lai JS	?	n.a.	+	+	?	+	?	Yes
Huang W, Li C	?	n.a.	+	+	+	+	+	Yes
Li J, Li M	?	n.a.	+	+	?	?	-	No
Xu J, Yang X	?	n.a.	+	+	?	+	?	Yes
Zou X, Li S	?	n.a.	+	+	-	+	-	Yes

B

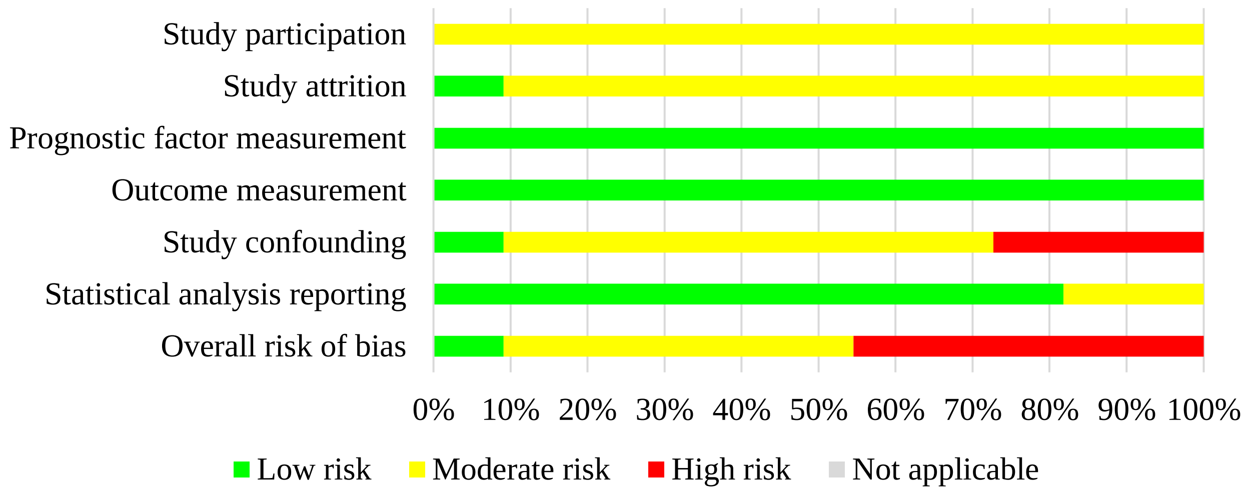

Supplementary Figure 3: Risk of bias assessment on study level [A] and across studies [B] comparing deceased and discharged critically ill patients with COVID-19

1: Assessed confounding factors are age, hypertension, heart failure and diabetes 2: As we analyzed raw data in the meta-analyses, statistical approaches of individual studies do no imply risk for this domain

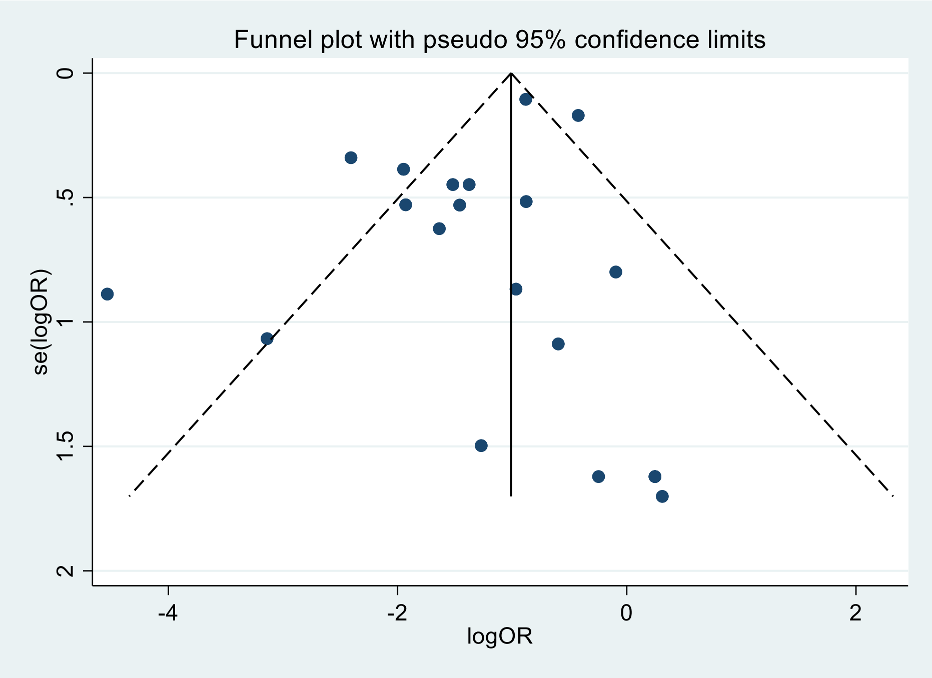

Supplementary Figure 4: Funnel plot of the studies reporting on mortality among all COVID-19 patients and baseline total white blood cell count. The visual assessment of the funnel plot and the Egger's test (p=0.134) did not indicate asymmetry and therefore small study effect is not likely to be present. Each dot represents a comparison. The y-axis is the standard error of the effect estimate. Larger studies with higher power are placed towards the top and lower powered studies are placed towards the bottom. The x-axis shows the result for the studies expressed in odds ratio.

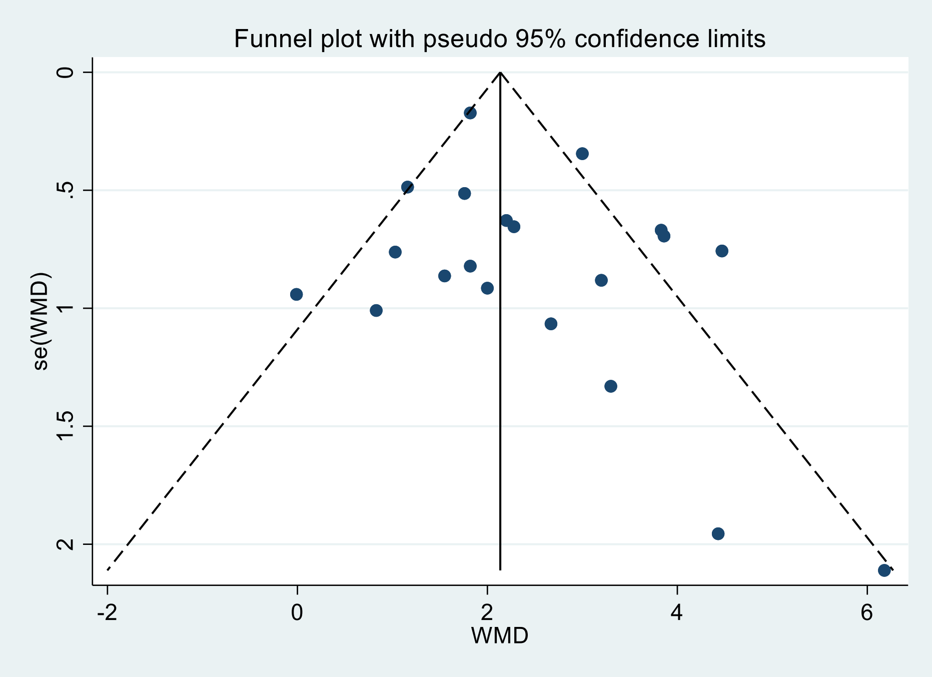

Supplementary Figure 5: Funnel plot of the studies reporting on mortality among all COVID-19 patients and baseline total white blood cell count. The visual assessment of the funnel plot and the Egger's test (p=0.196) did not indicate asymmetry and therefore small study effect is not likely to be present. Each dot represents a comparison. The y-axis is the standard error of the effect estimate. Larger studies with higher power are placed towards the top and lower powered studies are placed towards the bottom. The x-axis shows the result for the studies expressed in weighted mean difference.

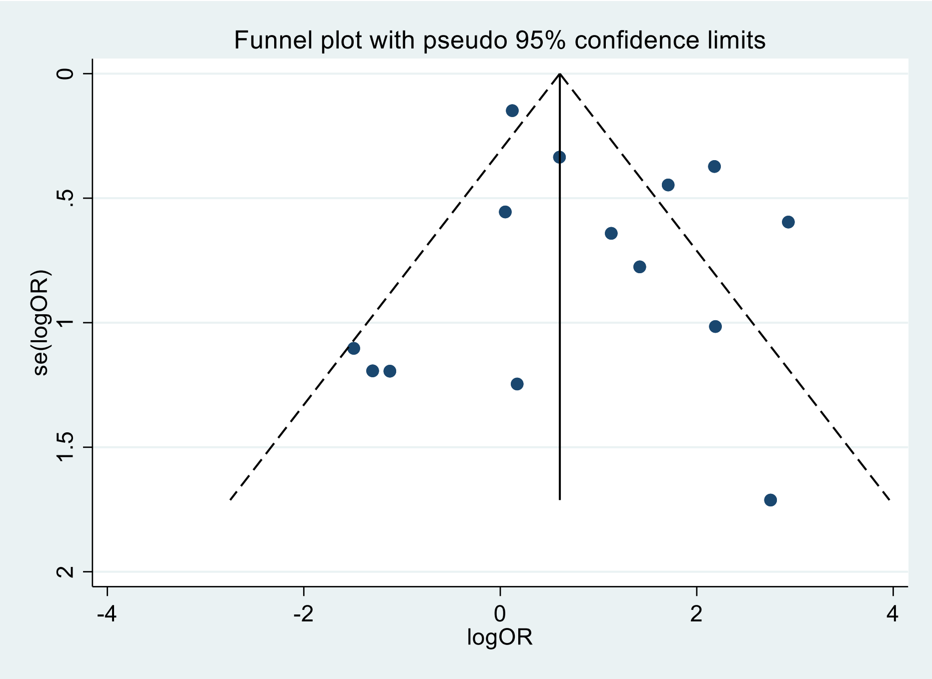

Supplementary Figure 6: Funnel plot of the studies reporting on mortality among all COVID-19 patients and baseline absolute lymphocyte count. The visual assessment of the funnel plot and the Egger's test (p=0.302) did not indicate asymmetry and therefore small study effect is not likely to be present. Each dot represents a comparison. The y-axis is the standard error of the effect estimate. Larger studies with higher power are placed towards the top and lower powered studies are placed towards the bottom. The x-axis shows the result for the studies expressed in odds ratio.

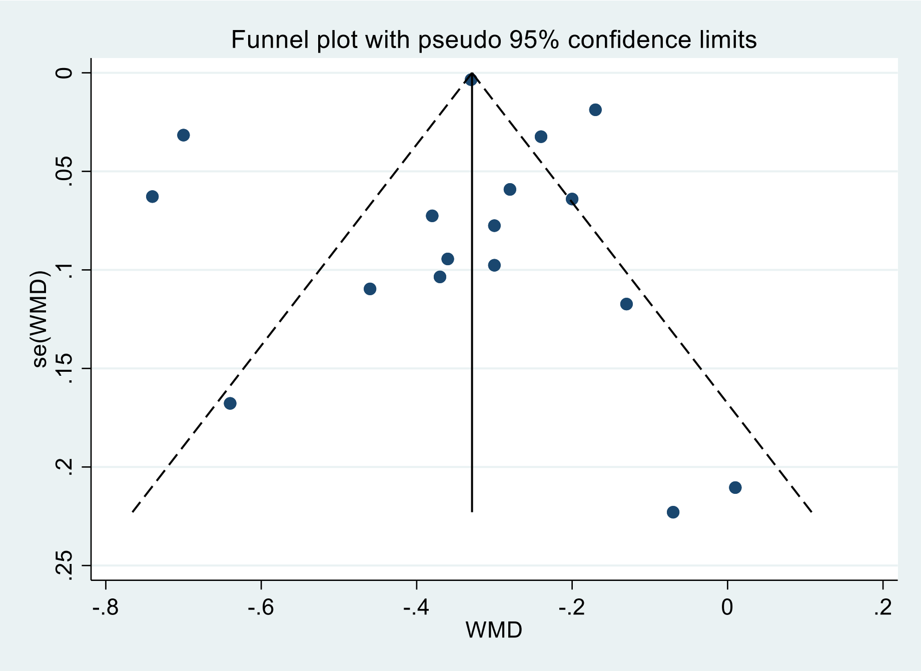

Supplementary Figure 7: Funnel plot of the studies reporting on mortality among all COVID-19 patients and baseline absolute lymphocyte count. The visual assessment of the funnel plot and the Egger's test (p=0.807) did not indicate asymmetry and therefore small study effect is not likely to be present. Each dot represents a comparison. The y-axis is the standard error of the effect estimate. Larger studies with higher power are placed towards the top and lower powered studies are placed towards the bottom. The x-axis shows the result for the studies expressed in weighted mean difference.

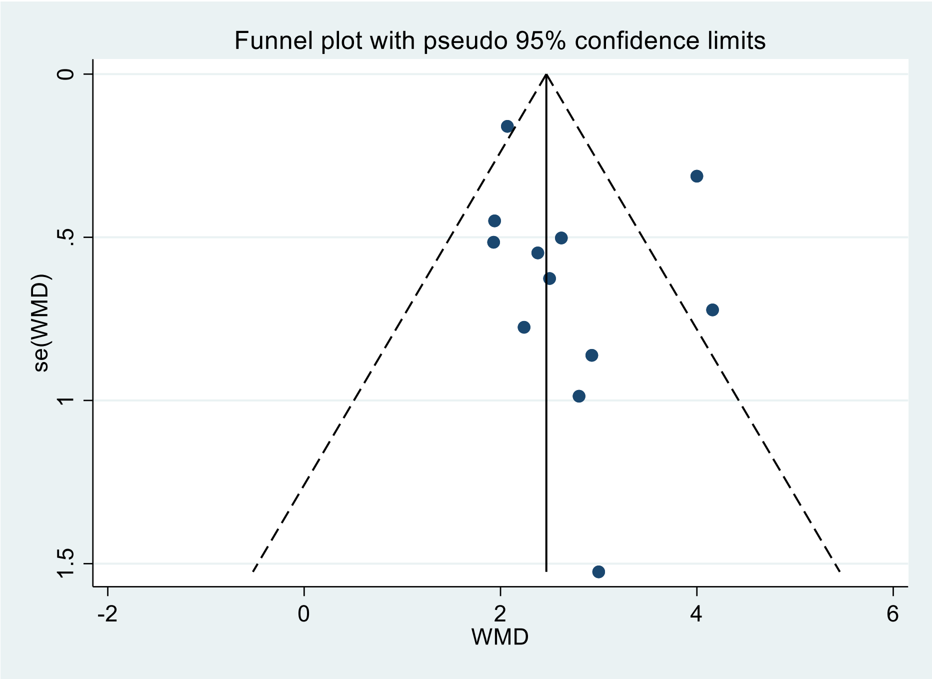

Supplementary Figure 8: Funnel plot of the studies reporting on mortality among all COVID-19 patients and baseline absolute neutrophil count The visual assessment of the funnel plot and the Egger's test (p=0.345) did not indicate asymmetry and therefore small study effect is not likely to be present. Each dot represents a comparison. The y-axis is the standard error of the effect estimate. Larger studies with higher power are placed towards the top and lower powered studies are placed towards the bottom. The x-axis shows the result for the studies expressed in weighted mean difference.

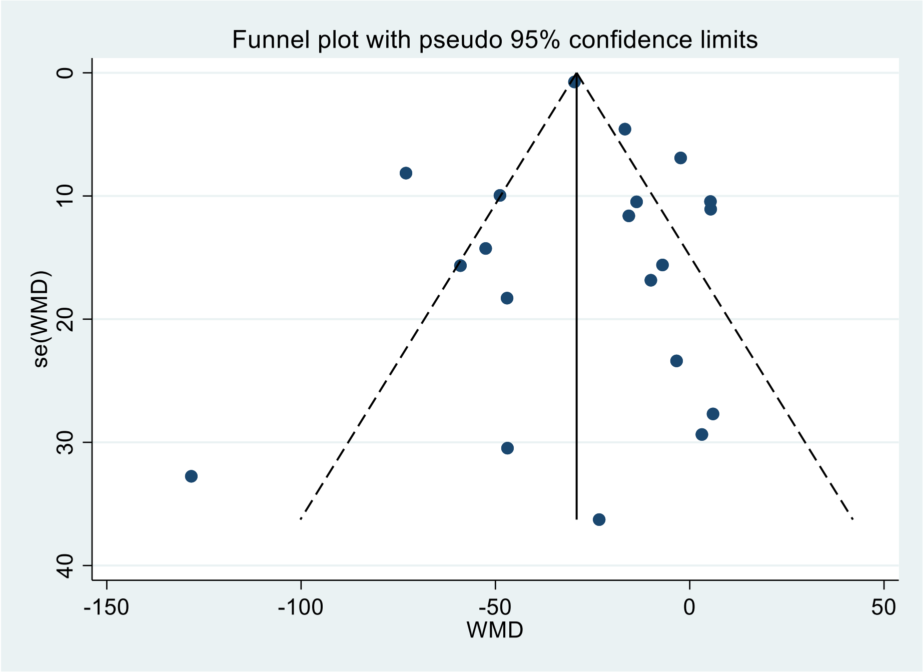

Supplementary Figure 9: Funnel plot of the studies reporting on mortality among all COVID-19 patients and baseline platelet count. The visual assessment of the funnel plot and the Egger's test (p=0.569) did not indicate asymmetry and therefore small study effect is not likely to be present. Each dot represents a comparison. The y-axis is the standard error of the effect estimate. Larger studies with higher power are placed towards the top and lower powered studies are placed towards the bottom. The x-axis shows the result for the studies expressed in weighted mean difference.

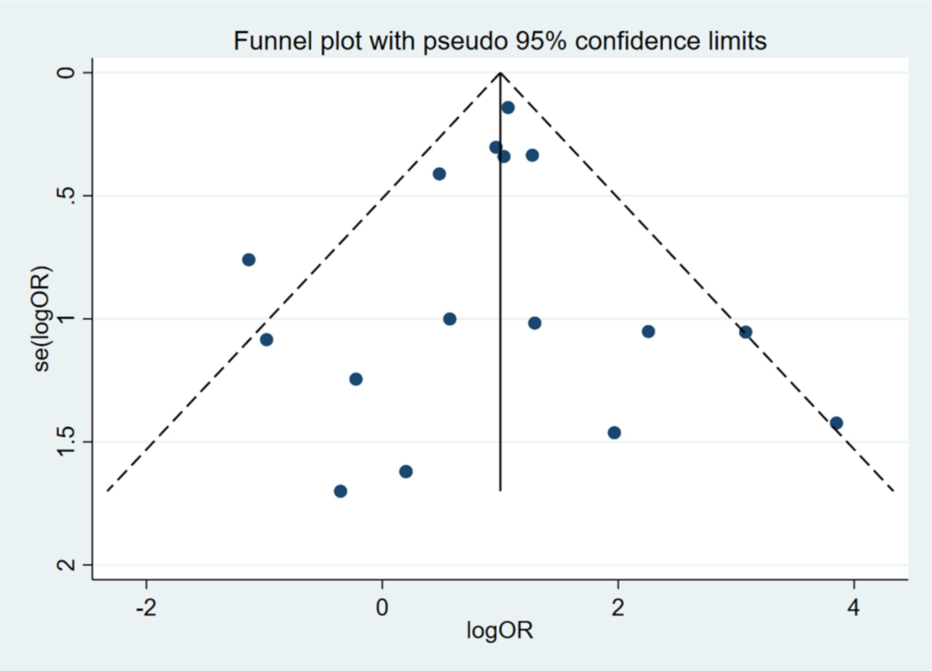

Supplementary Figure 10: Funnel plot of the studies reporting on mortality among all COVID-19 patients and baseline C-reactive protein. The visual assessment of the funnel plot and the Egger's test (p=0.649) did not indicate asymmetry and therefore small study effect is not likely to be present. Each dot represents a comparison. The y-axis is the standard error of the effect estimate. Larger studies with higher power are placed towards the top and lower powered studies are placed towards the bottom. The x-axis shows the result for the studies expressed in odds ratio.

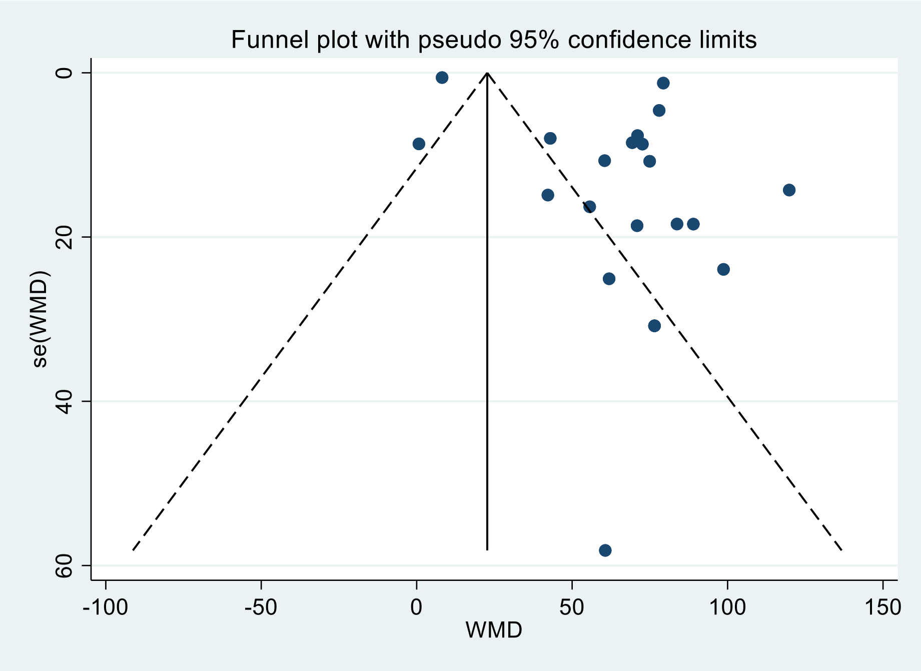

Supplementary Figure 11: Funnel plot of the studies reporting on mortality among all COVID-19 patients and C-reactive protein. The visual assessment of the funnel plot and the Egger's test (p=0.087) indicate asymmetry and therefore small study effect is likely to be present. Each dot represents a comparison. The y-axis is the standard error of the effect estimate. Larger studies with higher power are placed towards the top and lower powered studies are placed towards the bottom. The x-axis shows the result for the studies expressed in weighted mean difference.

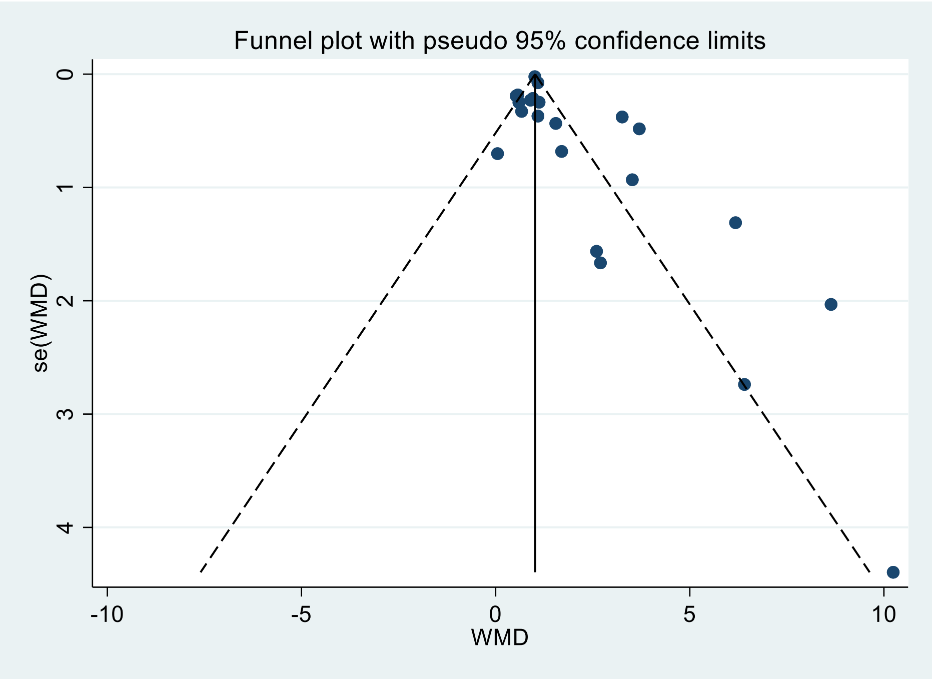

Supplementary Figure 12: Funnel plot of the studies reporting on mortality among all COVID-19 patients and baseline D-dimer. The visual assessment of the funnel plot and the Egger's test (p=0.037) indicate asymmetry and therefore small study effect is likely to be present. Each dot represents a comparison. The y-axis is the standard error of the effect estimate. Larger studies with higher power are placed towards the top and lower powered studies are placed towards the bottom. The x-axis shows the result for the studies expressed in weighted mean difference.

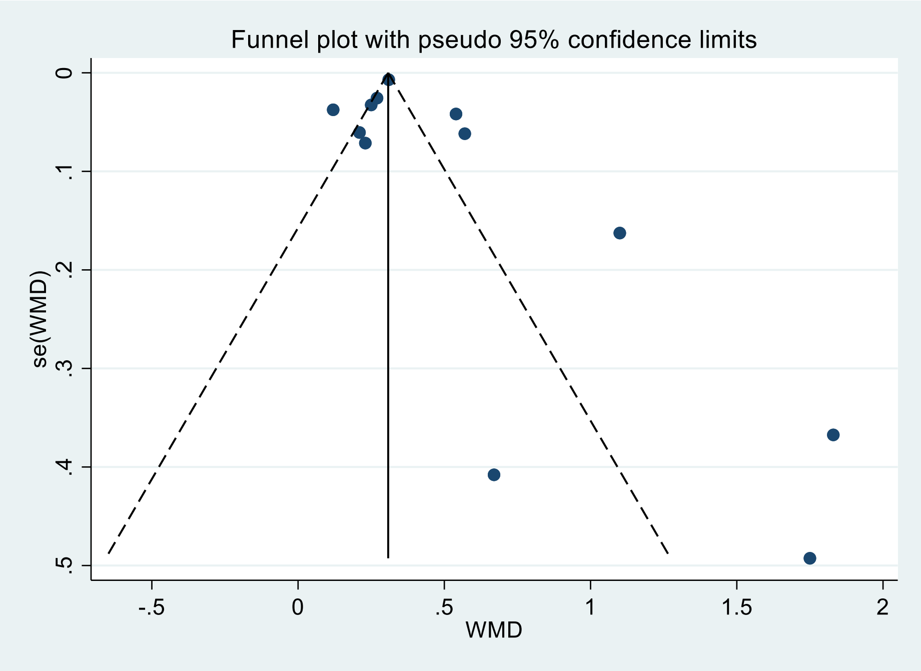

Supplementary Figure 13: Funnel plot of the studies reporting on mortality among all COVID-19 patients and baseline D-dimer. The visual assessment of the funnel plot and the Egger's test (p=0.005) indicate asymmetry and therefore small study effect is likely to be present. Each dot represents a comparison. The y-axis is the standard error of the effect estimate. Larger studies with higher power are placed towards the top and lower powered studies are placed towards the bottom. The x-axis shows the result for the studies expressed in odds ratio.

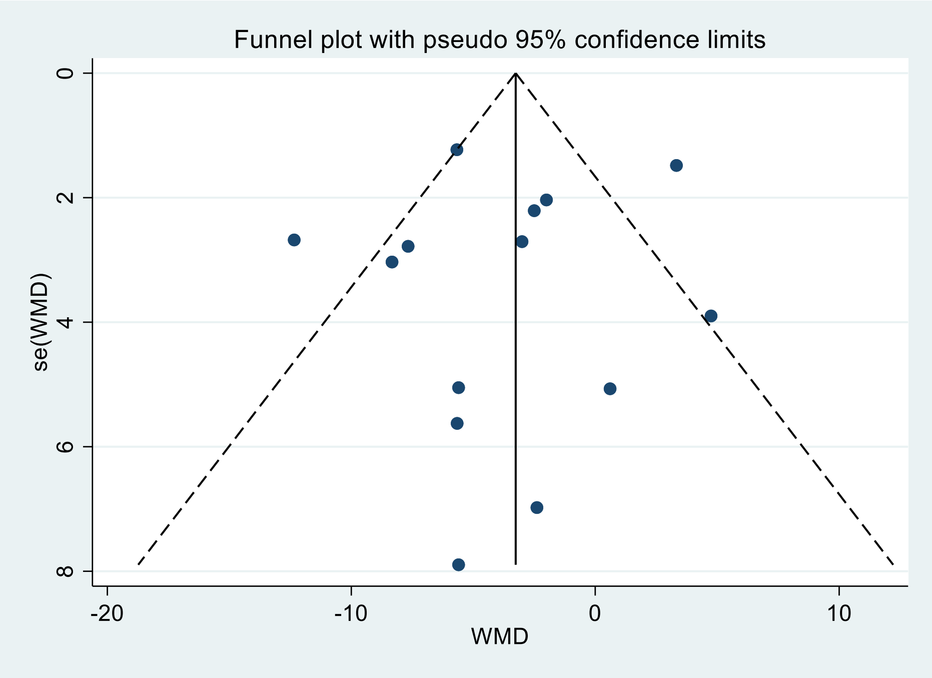

Supplementary Figure 14: Funnel plot of the studies reporting on mortality among all COVID-19 patients and baseline haemoglobin. The visual assessment of the funnel plot and the Egger's test (p=0.707) did not indicate asymmetry and therefore small study effect is not likely to be present. Each dot represents a comparison. The y-axis is the standard error of the effect estimate. Larger studies with higher power are placed towards the top and lower powered studies are placed towards the bottom. The x-axis shows the result for the studies expressed in weighted mean difference.

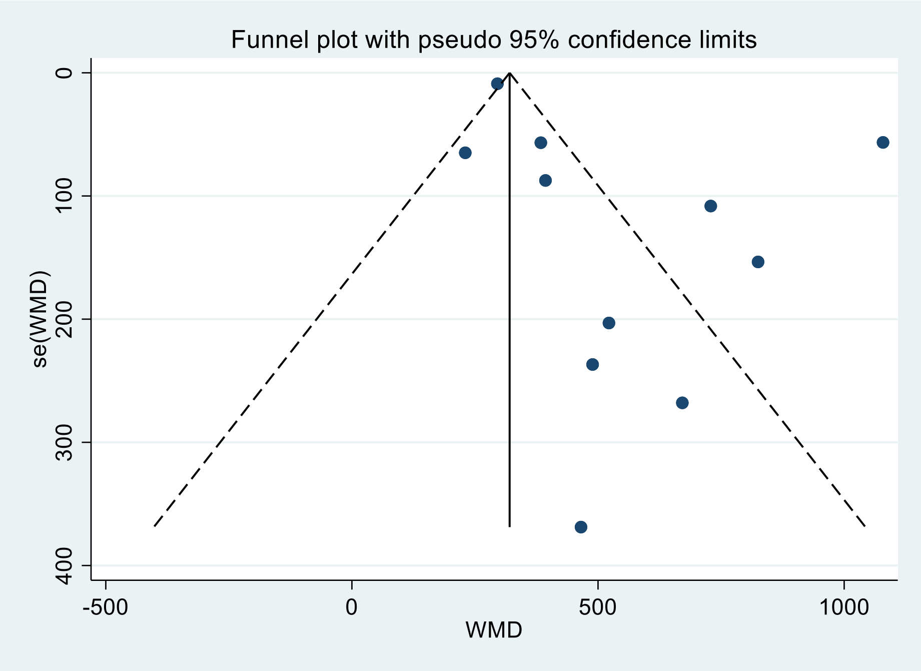

Supplementary Figure 15: Funnel plot of the studies reporting on mortality among all COVID-19 patients and baseline ferritin. The visual assessment of the funnel plot and the Egger's test (p=0.103) did not indicate asymmetry and therefore small study effect is not likely to be present. Each dot represents a comparison. The y-axis is the standard error of the effect estimate. Larger studies with higher power are placed towards the top and lower powered studies are placed towards the bottom. The x-axis shows the result for the studies expressed in weighted mean difference.

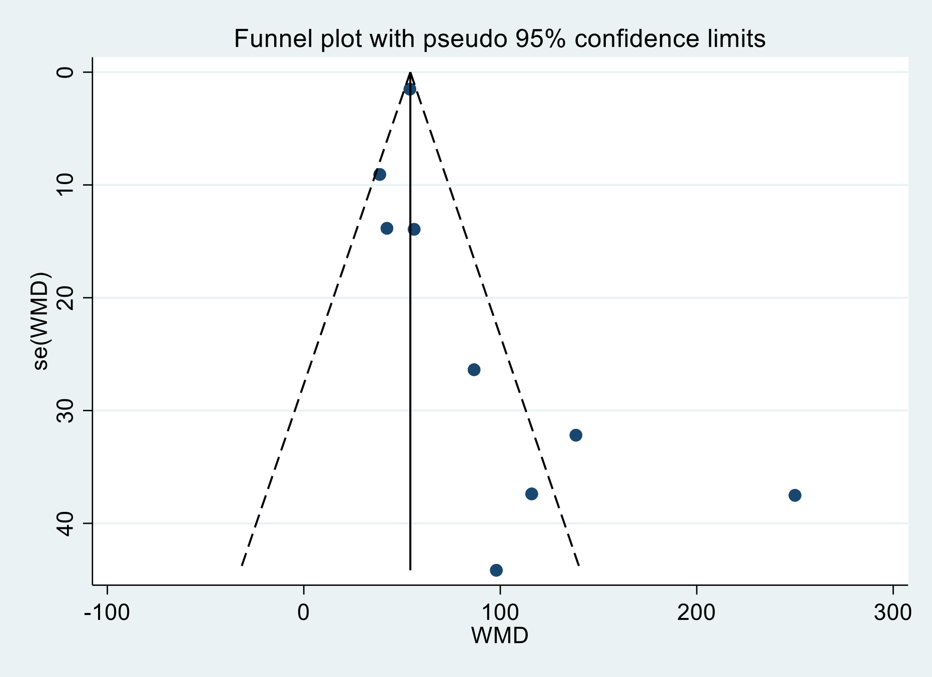

Supplementary Figure 16: Funnel plot of the studies reporting on mortality among all COVID-19 patients and creatine kinase. The visual assessment of the funnel plot and the Egger's test ($p<0.001$) indicate asymmetry and therefore small study effect is likely to be present. Each dot represents a comparison. The y-axis is the standard error of the effect estimate. Larger studies with higher power are placed towards the top and lower powered studies are placed towards the bottom. The x-axis shows the result for the studies expressed in weighted mean difference.

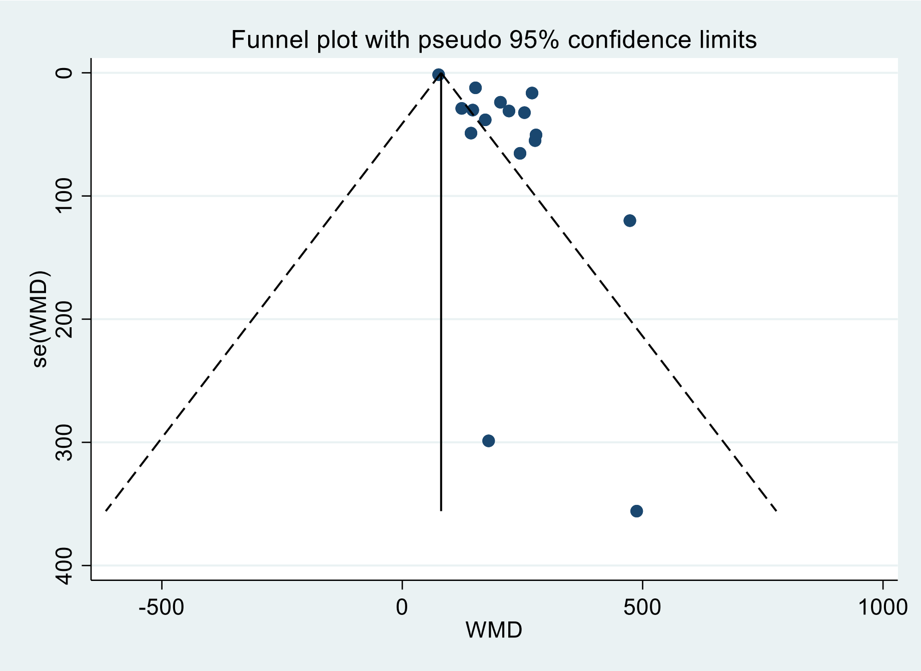

Supplementary Figure 17: Funnel plot of the studies reporting on mortality among all COVID-19 patients and baseline lactate dehydrogenase. The visual assessment of the funnel plot and the Egger's test ($p<0.001$) indicate asymmetry and therefore small study effect is likely to be present. Each dot represents a comparison. The y-axis is the standard error of the effect estimate. Larger studies with higher power are placed towards the top and lower powered studies are placed towards the bottom. The x-axis shows the result for the studies expressed in weighted mean difference.

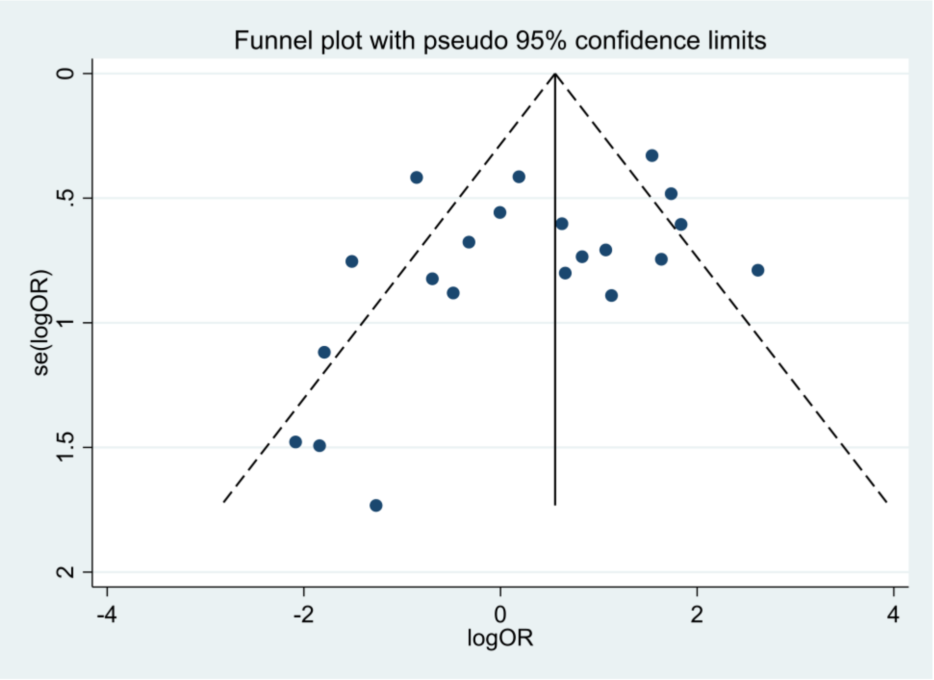

Supplementary Figure 18: Funnel plot of the studies reporting on instensive care requirement and baseline total white blood cell count. The visual assessment of the funnel plot and the Egger's test (p=0.124) did not indicate asymmetry and therefore small study effect is not likely to be present. Each dot represents a comparison. The y-axis is the standard error of the effect estimate. Larger studies with higher power are placed towards the top and lower powered studies are placed towards the bottom. The x-axis shows the result for the studies expressed in odds ratio.

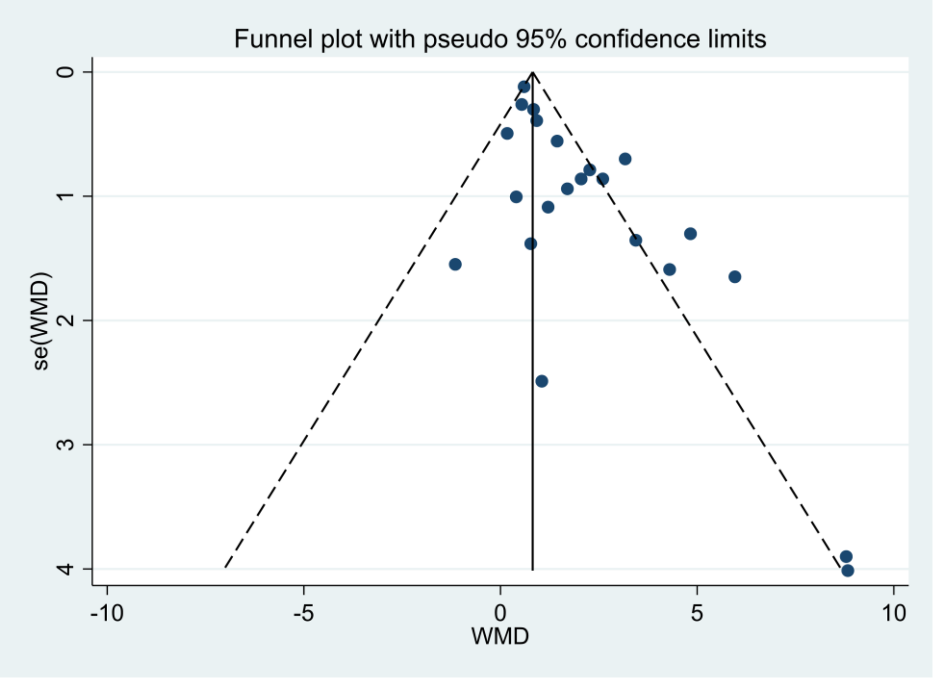

Supplementary Figure 19: Funnel plot of the studies reporting on mortality among all COVID-19 patients and baseline total white blood cell count. The visual assessment of the funnel plot and the Egger's test (p<0.001) indicate asymmetry and therefore small study effect is likely to be present. Each dot represents a comparison. The y-axis is the standard error of the effect estimate. Larger studies with higher power are placed towards the top and lower powered studies are placed towards the bottom. The x-axis shows the result for the studies expressed in weighted mean difference.

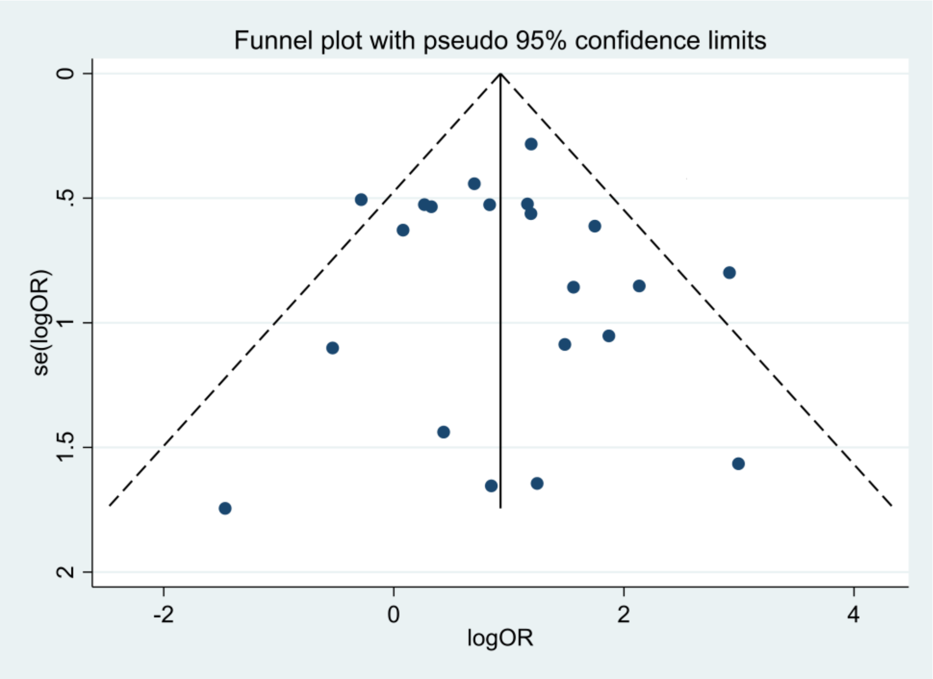

Supplementary Figure 20: Funnel plot of the studies reporting on instensive care requirement and baseline absolute lypmhocyte count. The visual assessment of the funnel plot and the Egger's test (p=0.738) did not indicate asymmetry and therefore small study effect is not likely to be present. Each dot represents a comparison. The y-axis is the standard error of the effect estimate. Larger studies with higher power are placed towards the top and lower powered studies are placed towards the bottom. The x-axis shows the result for the studies expressed in odds ratio.

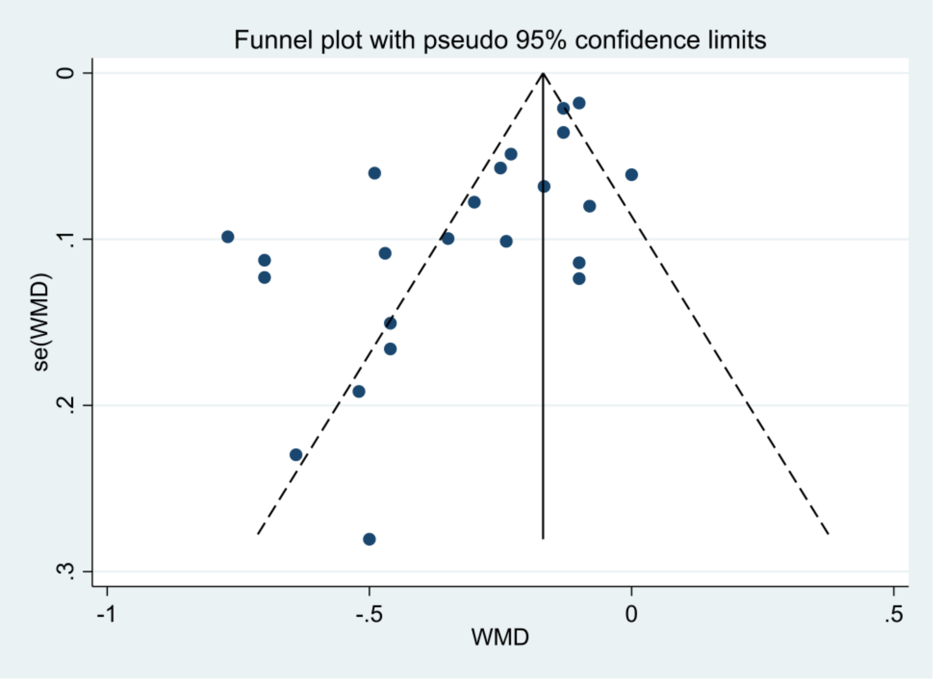

Supplementary Figure 21: Funnel plot of the studies reporting on mortality among all COVID-19 patients and baseline absolute lymphocyte count. The visual assessment of the funnel plot and the Egger's test (p<0.001) indicate asymmetry and therefore small study effect is likely to be present. Each dot represents a comparison. The y-axis is the standard error of the effect estimate. Larger studies with higher power are placed towards the top and lower powered studies are placed towards the bottom. The x-axis shows the result for the studies expressed in weighted mean difference.

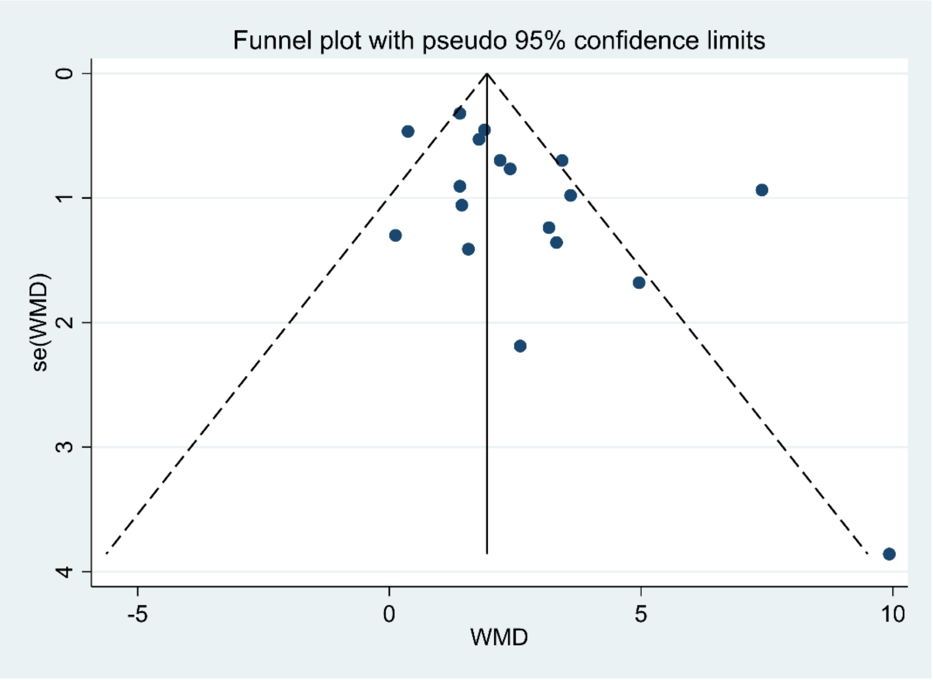

Supplementary Figure 22: Funnel plot of the studies reporting on instensive care requirement and baseline absolute neutrophil count. The visual assessment of the funnel plot and the Egger's test (p=0.037) indicate asymmetry and therefore small study effect is likely to be present. Each dot represents a comparison. The y-axis is the standard error of the effect estimate. Larger studies with higher power are placed towards the top and lower powered studies are placed towards the bottom. The x-axis shows the result for the studies expressed in weighted mean difference .

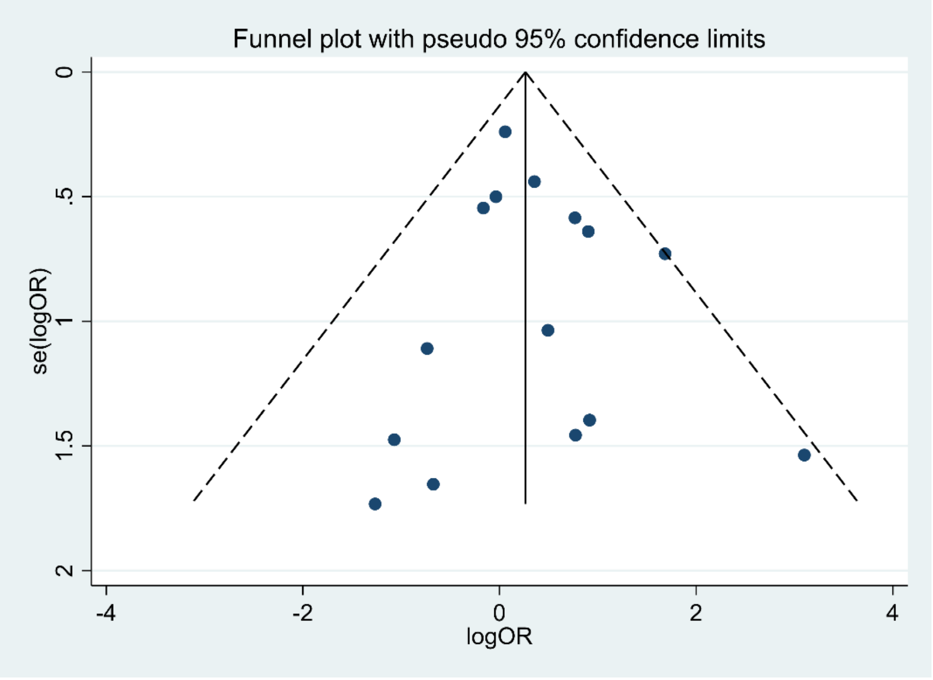

Supplementary Figure 23: Funnel plot of the studies reporting on mortality among all COVID-19 patients and platelet count. The visual assessment of the funnel plot and the Egger's test (p=0.410) did not indicate asymmetry and therefore small study effect is not likely to be present. Each dot represents a comparison. The y-axis is the standard error of the effect estimate. Larger studies with higher power are placed towards the top and lower powered studies are placed towards the bottom. The x-axis shows the result for the studies expressed in odds ratios.

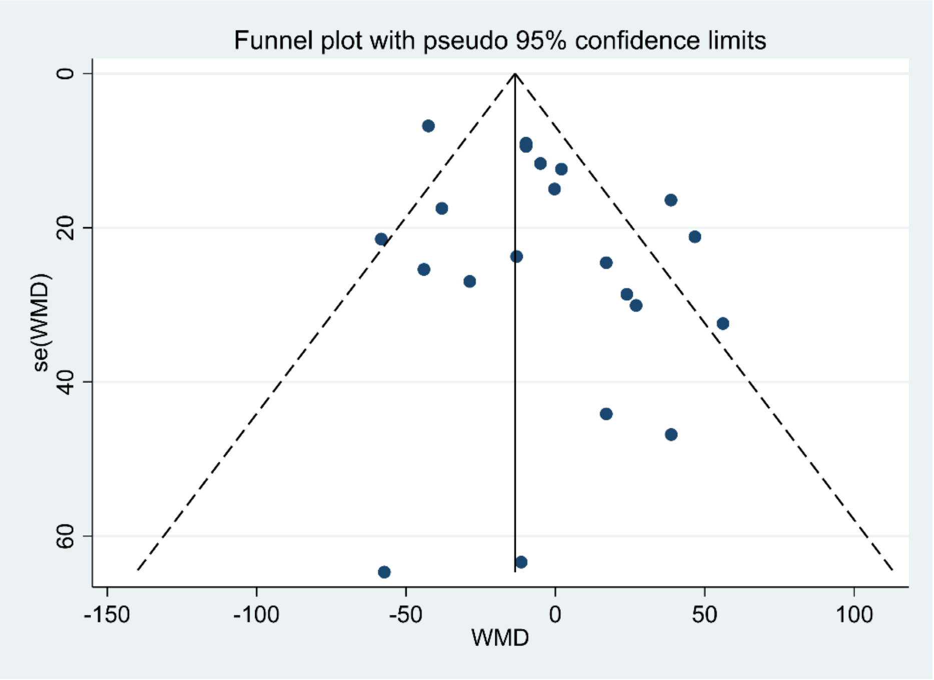

Supplementary Figure 24: Funnel plot of the studies reporting on instensive care requirement and baseline absolute platelet count. The visual assessment of the funnel plot and the Egger's test (p=0.075) indicate asymmetry and therefore small study effect is likely to be present. Each dot represents a comparison. The y-axis is the standard error of the effect estimate. Larger studies with higher power are placed towards the top and lower powered studies are placed towards the bottom. The x-axis shows the result for the studies expressed in weighted mean difference.

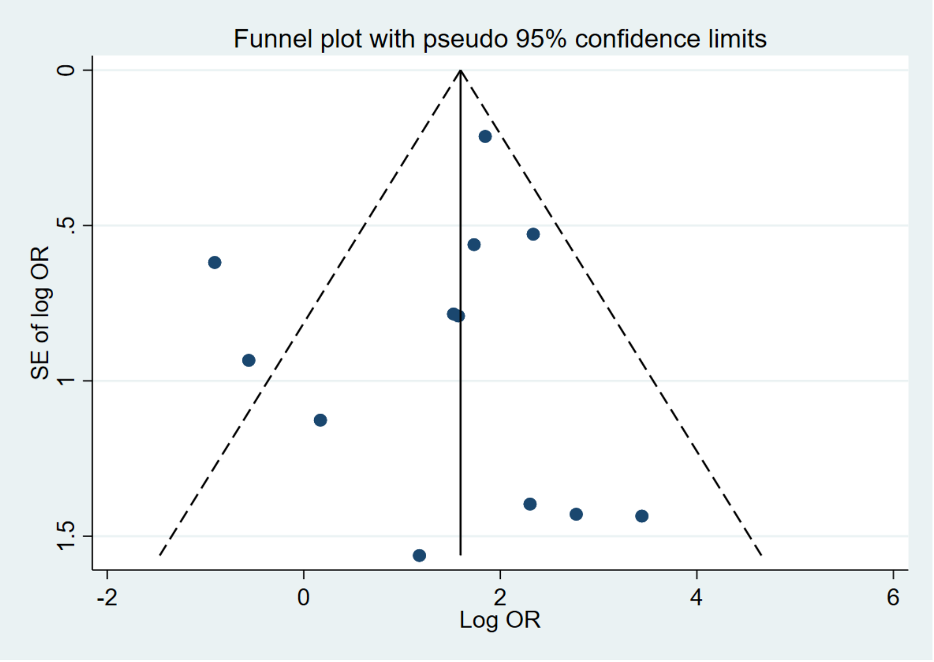

Supplementary Figure 25: Funnel plot of the studies reporting on mortality among all COVID-19 patients and baseline C-reactive protein. The visual assessment of the funnel plot and the Egger's test (p=0.474) did not indicate asymmetry and therefore small study effect is not likely to be present. Each dot represents a comparison. The y-axis is the standard error of the effect estimate. Larger studies with higher power are placed towards the top and lower powered studies are placed towards the bottom. The x-axis shows the result for the studies expressed in odds ratios.

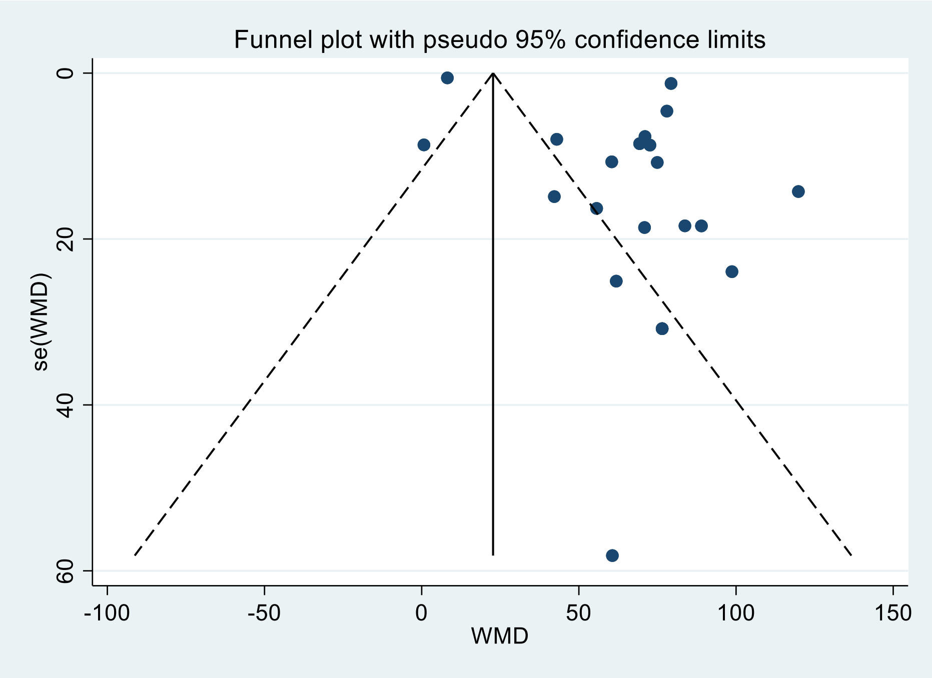

Supplementary Figure 26: Funnel plot of the studies reporting on instensive care requirement and baseline C-reactive protein. The visual assessment of the funnel plot and the Egger's test (p=0.059) indicate asymmetry and therefore small study effect is likely to be present. Each dot represents a comparison. The y-axis is the standard error of the effect estimate. Larger studies with higher power are placed towards the top and lower powered studies are placed towards the bottom. The x-axis shows the result for the studies expressed in weighted mean difference .

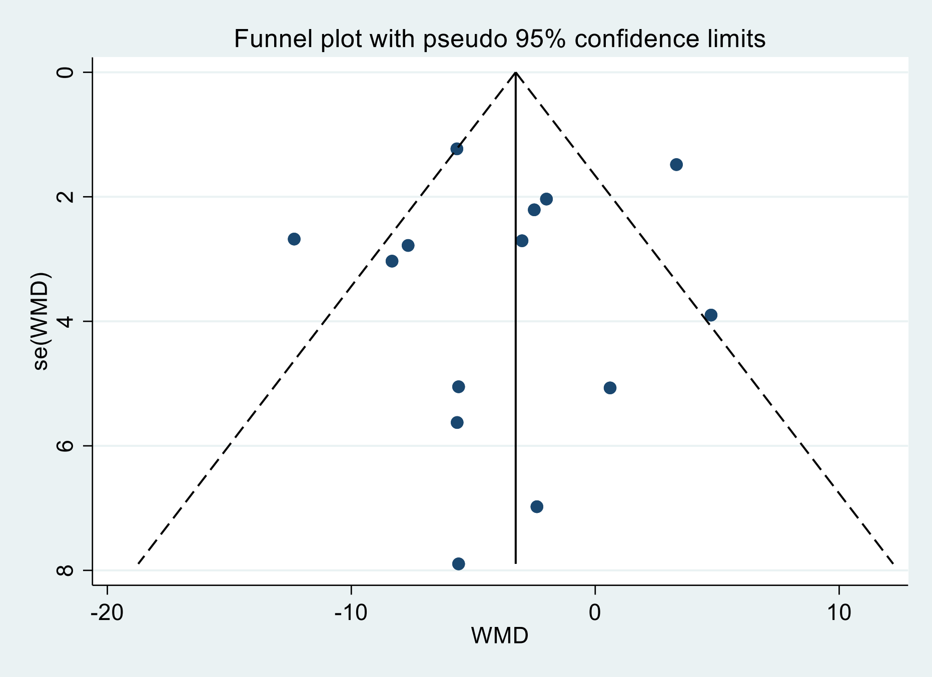

Supplementary Figure 27: Funnel plot of the studies reporting on instensive care requirement and baseline heamoglobin. The visual assessment of the funnel plot and the Egger's test (p=0.230) did indicate asymmetry and therefore small study effect is not likely to be present. Each dot represents a comparison. The y-axis is the standard error of the effect estimate. Larger studies with higher power are placed towards the top and lower powered studies are placed towards the bottom. The x-axis shows the result for the studies expressed in weighted mean difference .

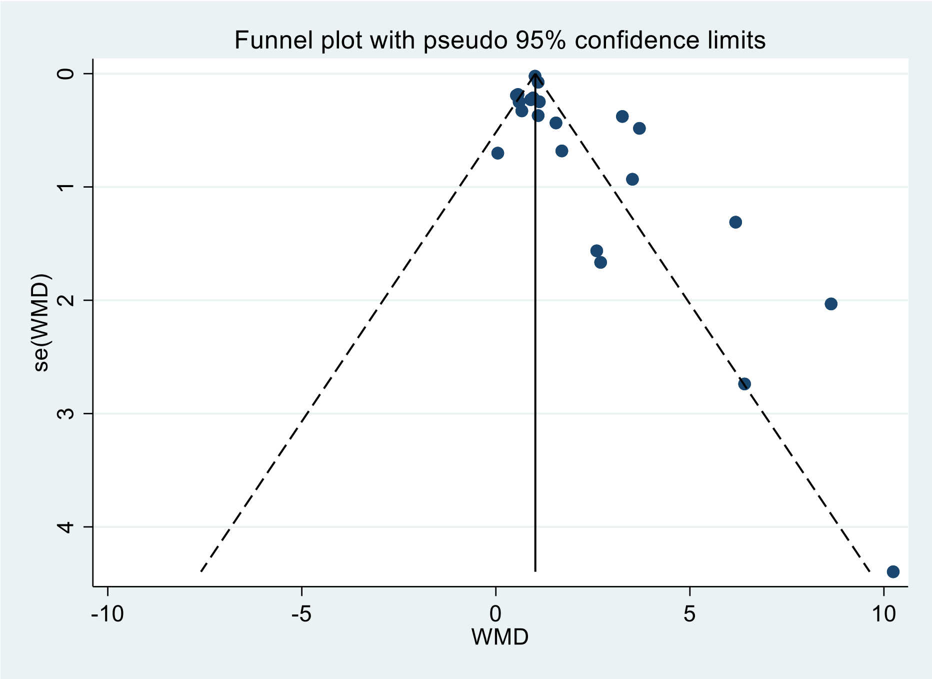

Supplementary Figure 28: Funnel plot of the studies reporting on instensive care requirement and baseline D-Dimer. The visual assessment of the funnel plot and the Egger's test (p=0.007) indicate asymmetry and therefore small study effect is likely to be present. Each dot represents a comparison. The y-axis is the standard error of the effect estimate. Larger studies with higher power are placed towards the top and lower powered studies are placed towards the bottom. The x-axis shows the result for the studies expressed in weighted mean difference .

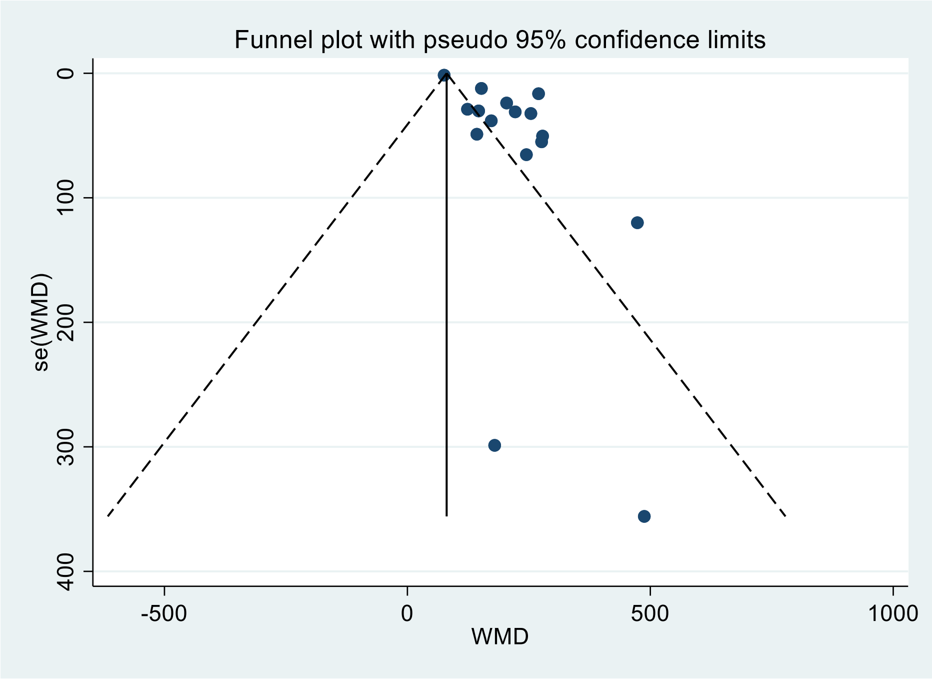

Supplementary Figure 29: Funnel plot of the studies reporting on instensive care requirement and baseline lactate dehydrogenase. The visual assessment of the funnel plot and the Egger's test (p=0.141) did indicate asymmetry and therefore small study effect is not likely to be present. Each dot represents a comparison. The y-axis is the standard error of the effect estimate. Larger studies with higher power are placed towards the top and lower powered studies are placed towards the bottom. The x-axis shows the result for the studies expressed in weighted mean difference.

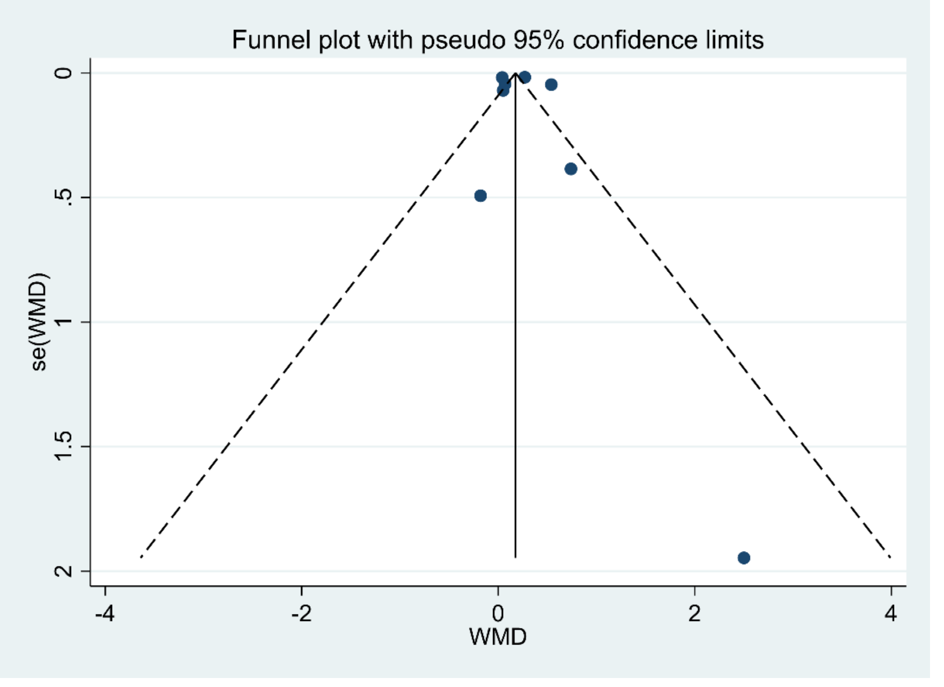

Supplementary Figure 30: Funnel plot of the studies reporting on instensive care requirement and baseline procalcitonin. The visual assessment of the funnel plot and the Egger's test (p=0.735) did not indicate asymmetry and therefore small study effect is not likely to be present. Each dot represents a comparison. The y-axis is the standard error of the effect estimate. Larger studies with higher power are placed towards the top and lower powered studies are placed towards the bottom. The x-axis shows the result for the studies expressed in weighted mean difference.

PRISMA 2009 Checklist

Section/topic	#	Checklist item	Reported on page #
**TITLE**			
Title	1	Identify the report as a systematic review, meta-analysis, or both.	Page 1
**ABSTRACT**			
Structured summary	2	Provide a structured summary including, as applicable: background; objectives; data sources; study eligibility criteria, participants, and interventions; study appraisal and synthesis methods; results; limitations; conclusions and implications of key findings; systematic review registration number.	Page 2
**INTRODUCTION**			
Rationale	3	Describe the rationale for the review in the context of what is already known.	Page 4
Objectives	4	Provide an explicit statement of questions being addressed with reference to participants, interventions, comparisons, outcomes, and study design (PICOS).	Page 4
**METHODS**			
Protocol and registration	5	Indicate if a review protocol exists, if and where it can be accessed (e.g., Web address), and, if available, provide registration information including registration number.	Page 4
Eligibility criteria	6	Specify study characteristics (e.g., PICOS, length of follow-up) and report characteristics (e.g., years considered, language, publication status) used as criteria for eligibility, giving rationale.	Page 4–5
Information sources	7	Describe all information sources (e.g., databases with dates of coverage, contact with study authors to identify additional studies) in the search and date last searched.	Page 4
Search	8	Present full electronic search strategy for at least one database, including any limits used, such that it could be repeated.	Page 4
Study selection	9	State the process for selecting studies (i.e., screening, eligibility, included in systematic review, and, if applicable, included in the meta-analysis).	Page 4
Data collection process	10	Describe method of data extraction from reports (e.g., piloted forms, independently, in duplicate) and any processes for obtaining and confirming data from investigators.	Page 5
Data items	11	List and define all variables for which data were sought (e.g., PICOS, funding sources) and any assumptions and simplifications made.	Page 5
Risk of bias in individual studies	12	Describe methods used for assessing risk of bias of individual studies (including specification of whether this was done at the study or outcome level), and how this information is to be used in any data synthesis.	Page 5
Summary measures	13	State the principal summary measures (e.g., risk ratio, difference in means).	Page 5
Synthesis of results	14	Describe the methods of handling data and combining results of studies, if done, including measures of consistency (e.g., I^2) for each meta-analysis.	Page 5

PRISMA 2009 Checklist

Section/topic	#	Checklist item	Reported on page #
Risk of bias across studies	15	Specify any assessment of risk of bias that may affect the cumulative evidence (e.g., publication bias, selective reporting within studies).	Page 5
Additional analyses	16	Describe methods of additional analyses (e.g., sensitivity or subgroup analyses, meta-regression), if done, indicating which were pre-specified.	Not applicable
**RESULTS**			
Study selection	17	Give numbers of studies screened, assessed for eligibility, and included in the review, with reasons for exclusions at each stage, ideally with a flow diagram.	Page 5<br>Figure 1
Study characteristics	18	For each study, present characteristics for which data were extracted (e.g., study size, PICOS, follow-up period) and provide the citations.	Suppl. Table 1
Risk of bias within studies	19	Present data on risk of bias of each study and, if available, any outcome level assessment (see item 12).	Suppl. Figure 1–3
Results of individual studies	20	For all outcomes considered (benefits or harms), present, for each study: (a) simple summary data for each intervention group (b) effect estimates and confidence intervals, ideally with a forest plot.	Figure 2-3<br>Suppl. Table 4
Synthesis of results	21	Present results of each meta-analysis done, including confidence intervals and measures of consistency.	Page 5–7<br>Suppl. Table 2–3
Risk of bias across studies	22	Present results of any assessment of risk of bias across studies (see Item 15).	Suppl. Figure 4–30
Additional analysis	23	Give results of additional analyses, if done (e.g., sensitivity or subgroup analyses, meta-regression [see Item 16]).	Not applicable
**DISCUSSION**			
Summary of evidence	24	Summarize the main findings including the strength of evidence for each main outcome; consider their relevance to key groups (e.g., healthcare providers, users, and policy makers).	Page 7–9
Limitations	25	Discuss limitations at study and outcome level (e.g., risk of bias), and at review-level (e.g., incomplete retrieval of identified research, reporting bias).	Page 9
Conclusions	26	Provide a general interpretation of the results in the context of other evidence, and implications for future research.	Page 9
**FUNDING**			
Funding	27	Describe sources of funding for the systematic review and other support (e.g., supply of data); role of funders for the systematic review.	Page 1

From: Moher D, Liberati A, Tetzlaff J, Altman DG, The PRISMA Group (2009). Preferred Reporting Items for Systematic Reviews and Meta-Analyses: The PRISMA Statement. PLoS Med 6(7): e1000097. doi:10.1371/journal.pmed1000097

For more information, visit: **www.prisma-statement.org**.
